# One-Particle Representation of Heat Conduction Described within the Scope of the Second Law

**DOI:** 10.1371/journal.pone.0145026

**Published:** 2016-01-13

**Authors:** Christopher Gunaseelan Jesudason

**Affiliations:** Chemistry Department and Center for Theoretical and Computation Physics, University of Malaya, 50603 Kuala Lumpur, Malaysia; China University of Mining and Technology, CHINA

## Abstract

The Carnot cycle and its deduction of maximum conversion efficiency of heat inputted and outputted isothermally at different temperatures necessitated the construction of isothermal and adiabatic pathways within the cycle that were mechanically “reversible”, leading eventually to the Kelvin-Clausius development of the entropy function S with differential dS=dq/T such that $\oint_{C}dS=0$ where the heat absorption occurs at the isothermal paths of the elementary Carnot cycle. Another required condition is that the heat transfer processes take place infinitely slowly and “reversibly”, implying that rates of transfer are not explicitly featured in the theory. The definition of ‘heat’ as that form of energy that is transferred as a result of a temperature difference suggests that the local mode of transfer of “heat” in the isothermal segments of the pathway implies a Fourier-like heat conduction mechanism which is apparently irreversible, leading to an increase in entropy of the combined reservoirs at either end of the conducting material, and which is deemed reversible mechanically. These paradoxes are circumvented here by first clarifying the terms used before modeling heat transfer as a thermodynamically reversible but mechanically irreversible process and applied to a one dimensional atomic lattice chain of interacting particles subjected to a temperature difference exemplifying Fourier heat conduction. The basis of a “recoverable trajectory” i.e. that which follows a zero entropy trajectory is identified. The Second Law is strictly maintained in this development. A corollary to this zero entropy trajectory is the generalization of the Zeroth law for steady state non-equilibrium systems with varying temperature, and thus to a statement about “equilibrium” in steady state non-thermostatic conditions. An energy transfer rate term is explicitly identified for each particle and agrees quantitatively (and independently) with the rate of heat absorbed at the reservoirs held at different temperatures and located at the two ends of the lattice chain in MD simulations, where all energy terms in the simulation refer to a single particle interacting with its neighbors. These results validate the theoretical model and provides the necessary boundary conditions (for instance with regard to temperature differentials and force fields) that thermodynamical variables must comply with to satisfy the conditions for a recoverable trajectory, and thus determines the solution of the differential and integral equations that are used to model these processes. These developments and results, if fully pursued would imply that not only can the Carnot cycle be viewed as describing a local process of energy-work conversion by a single interacting particle which feature rates of energy transfer and conversion not possible in the classical Carnot development, but that even irreversible local processes might be brought within the scope of this cycle, implying a unified treatment of thermodynamically (i) irreversible (ii) reversible (iii) isothermal and (iv) adiabatic processes by conflating the classically distinct concept of work and heat energy into a single particle interactional process. A resolution to the fundamental and long-standing conjecture of Benofy and Quay concerning the Fourier principle is one consequence of the analysis.

## Introduction

A major objective of this work is to focus on heat conduction as it directly relates to the formulation of the Second law [1] where the definition of heat is concerned, and the implications of strictly conductive heat flow on how systems may be described by reference to such a phenomenon. General considerations taking into account conductive heat lead to rigorous theorems that describe conditions where various minimum entropy principles obtained under stated conditions, (Chap. IV [2], Sec. 3.2 [3]) which revealed that the much cited Prigogine derivations (see esp. p.76, [4]) are approximations at best (p.17–18, par. after Eq (55) [3]) and where these minimum principles need not necessarily obtain. A careful restatement of conductive heat based on the Benofy and Quay (BQ) study and analysis of heat conduction [5] lead to a description of any steady state system (fluid phase in particular) with a temperature gradient with the fluxes obeying Onsager reciprocity conditions to any order (Sec.4, Eqs (49–54), Appendix, Eqs (A3–A6) [6]) without the use of the cardinal time-reversibility arguments which was proven to be a mathematically inappropriate and frequently incorrect concept when applied to physical systems [7, 8]. Other illustrations of conductive heat include the theory of thermal desorption of gases from a surface (sec. 4.4, Eqs 84–98 [9], Eqs 1–44 [10]) where the modern forms stem from the foundations laid by Redhead [11], where it is shown that for disintegrating systems, there is a coordinate trajectory along which the entropy is conserved; such trajectories are termed “recoverable” [9] which extends the Redhead analysis. A new formulation of statistical mechanics for such disintegrating systems had to be constructed for such non-equilibrium (NE) and irreversible phenomena (Sec. 3, Eqs 24–32 [9]). This development could be written in variational form for the class used to derive the more precise form of the Kelvin relations (Eq 1 [12]) as

$$\begin{array}{c} \delta {\left[\int_{\partial C}dS\right]}_{U,\{x\}}=0 \end{array}$$ (1)

where the (Euler-Lagrange) variation of the entropy *S* is not over the system but the trajectory ∂*C* of a “macroparticle” subject to the constraints of fixed energy content *U* and external constraints {*x*} pertinent to the entire system, where the standard Kelvin identities appear as first order relations (Sec. 3, Eq 18–19 [12]). Liouville spaces are not used in the trajectory description (p.169, top par. [9]). In the above description, the system is in the steady state with “work-heat” interconversion where the usual partition of work and heat is maintained. Thus one interesting but not obvious question to pose given the above sequence of deductions concerning work-heat transformations would be whether “pure” heat conduction, as a phenomenon described by the Second law as leading to an increase in entropy when conveyed between two heat reservoirs can in fact be decomposed into a series of interactions that in fact imply conformance to the Second law at the local level—as with the above examples of work-heat optimal transformations—whilst maintaining optimal efficiency relative to the moving frame of reference used, without any violation of the Second law as it is currently understood, especially in relation to fixed frames of reference? This question also reverses the very involved discussion of BQ concerning the Second law (p.10 last par.- p.11, first par. [5]), Fourier heat conduction *and* their theory of Thermomagnetics where the concept of “locality” seems to be associated with non-work transitions, i.e. pure heat transfer without work (p.11 [5]). BQ have defined the Fourier principle **F** for all practical purposes as embodied in the inequality **q**.∇*T* ≤ 0 where **q** should be interpreted as solely the heat conduction current with *T* the temperature:

“On the other hand, as seen above, **F** (*the Fourier principle*) is a strictly local principle that cannot be applied as it stands to systems as a whole but holds for every point of a system and does not permit local exceptions based on the behavior of the rest of the system. No matter what happens in the rest of the system, it is the local temperature-gradient that determines, along with the material properties, the direction of heat-conduction at a given point.” (Italics mine)

Such a strict partitioning is also associated with zero work, if all the energy transferred is due to conduction, i.e. heat energy transfer. Clearly this viewpoint is adopted whenever the First law is expressed as

$$\begin{array}{c} dU=dq+dW \end{array}$$ (2)

with the energy *dU* composed of increments due to heat *dq* and work *dW*, where these two increments are strictly orthogonal and do not share the same function space.

Incidentally, it was shown through a rigorous contrapositive argument that the BQ condition (akin to their understanding of **F**) for all steady state systems ∫_*V*_ ∇ ⋅ **J**_*q*_/*T*)*dV* ≤ 0 [13] had to be replaced by ∫_*V*_ ∇ ⋅ (**J**_*q*_/*T*)*dV* ≤ 0 (Eq 13 [14], p.55, Eq (13) [2]), which lead to a correction −*ε*∇*T* in their thermoelectric equation (Eq 13 [15]) from their expression ∇*ϕ* = *τ*∇*T* (Eq 14 [15]). Also, using the corrected expression lead, by expansion of terms, to a different description of thermoelectricity where the Kelvin identities are first order approximations [12], where in a moving frame, the equality of the corrected expression obtains for a defined recoverable trajectory. For instance, (p.12, Eq (34(a-b), second citation, p.15 [12, 16]) the expressions for the Seebeck coefficient (with *σ*_*T*, *c*_ = 0, *T* = 0) and without Nernst law assumptions is

$$\varepsilon_{T^{\prime },c}=\left[\overset{T^{\prime }}{\underset{0}{\int }}\sigma_{T,c}\;dT\right]/T^{\prime }$$ (3)

and

$$\varepsilon_{T^{"},c}T^{"}-\varepsilon_{T^{\prime },c}T^{\prime }=\int_{T\prime }^{T"}\sigma_{T,c}\;dT$$ (4)

respectively, where the *T*’s are the temperatures, the *ε*’s the Seebeck coefficient and the *σ*’s are the absolute Seebeck coefficient for current *I* = *c*. This may be contrasted to the (approximate) Kelvin form

$$\begin{array}{c} S_{2}-S_{1}=\int_{T_{1}}^{T_{2}}\left(\mu /T\right) \end{array}$$ (5)

with the *S*’s being the Seebeck coefficient according to Kelvin, and *μ* the Thomson coefficient. For BQ then, Fourier heat conduction is a strictly local phenomenon, whereas the Second law with the Carnot optimization refers to global boundary conditions, where local violations (e.g. the movement of “heat” energy from a colder to a hotter region) can occur if there is a global compensating mechanism (for instance, a Carnot device working in reverse transporting heat from a colder to a hotter thermal reservoir, where work must be performed on the system) so that in a complete analysis, no violation of the Second law would take place. Whilst it stands to reason that subject to the Second law being true, this must necessarily be the case, there seems to be a paradox in this approach in that it presupposes some type of super-memory effect where for any arrangement of the system, the various heat and work terms would adjust the efficiency in such a manner that there is no violation of the Carnot maximum efficiency for net transfer about two thermal reservoirs with differing temperatures; if not some remarkable memory effect over any arbitrary configuration of the system, then a non-random long-ranging force field must be posited for any arbitrary system such that however improbable, no violation of the Carnot efficiency would ensue. Since such a hypothesis is improbable, we attempt here to construct a paradigm whereby heat (as defined by energy transfer due solely to a temperature difference) is both analytically global and local in scope so instead of assuming the BQ partitioning of processes that are deemed to be either analytically global or local in nature, however improbable, it was decided to construct a paradigm whereby heat (defined as a form of energy transfer due solely to a temperature difference between two regions of a homogeneous material) was both analytically global and local in scope within the limits set by the atomic nature of matter; this implies that the force interactions would be interpreted as having energetics that are defined in both mechanical and thermodynamical terms locally. Since previous work had developed a paradigm of thermal desorption where the temperature of the molecule on the substrate surface is higher than the free molecules with zero entropy change for the moving ensemble of particles as it leaves the surface (Sec. 3 Development of Kinetic Postulates, Sec. 4.4 [9]), it was considered possible to extend this model to heat conduction as well, if one could account for the fact that in normal heat conduction, the system is not disintegrating, unlike the disintegrating case of a system of a fixed number of molecules leaving the surface. For a disintegrating system a theorem was proved (p. 177, Sec. 4.4 [9]) which asserted the following:

“If the generalized Carnot engine is disintegrating, then it is necessarily a frictionless device”.

The method used for a non-disintegrating system is to design a mechanism whereby one could consider scattering on several equally spaced lattice planes; if each lattice plane had a recoverable (zero entropy) trajectory, then the sum of all the scattering would also be recoverable. Thus this mechanism is both local, and at the same time global in terms of the Carnot efficiency. We believe such an analysis is more realistic as it does not involve the assumption of improbable events as discussed above. Since the above shows that many irreversible processes could be reduced to one that was equivalent to a conductive process, or to a recoverable system (as with thermocouples), then such a paradigm could conceivably simplify thermodynamical analyses, apart from providing a theoretical interpretation that could encourage the framing of the thermodyanamical equations for the First and Second laws in terms of such transitions about moving frames where the work and heat terms are not viewed as orthogonal and separable. One simplification that does away for instance of the need for time reversibility in developing reciprocity relations was the manner in which a steady state system within a temperature field was reduced to a strictly conducting system [6, 8, 17], where all the fluxes induced by the forces have a conductive component; other potential simplifications might result from the theory that draws attention to the nature of heat transport as a potential source of work without contradicting the Second law. As mentioned above, the problem one faces in forming this paradigm is that previous work focused on developing the recoverable or zero entropy trajectory for unidirectional irreversible scattering of particles under a mean potential $\bar{W}\left(r\right)$ (Eqs 93–98 [9]), whereas for basic steady state heat conduction processes involving a lattice chain—the model used here—there is also net back-scattering that must be accounted for and so appropriate local terms have to be identified, so that a net recoverable trajectory results. One spin-off from this paradigm is the generalization of the Zeroth law for a temperature non-invariant steady state system.

As with all paradigms—especially those that are more recently mooted—the elaboration of any of the details of the method outlined are protracted, often multi-generational intense research endeavors: the research questions that would arise might include the following:

1. how does the reference model adopted here for bounded particles translate for systems with particles in a free fluid molecular environment?
2. how does one deconvolve the various dynamics of the subspaces associated with the motion from an averaged motion?
3. what type of density distribution function(s) might be expected from the energy transfer mechanism and what is the relationship of the distribution function to the overall dynamics?

Questions such as the above for more complex systems would be topics addressed for future work, as the concepts introduced become more clear in its scope in time with further elaboration by various workers. However, in the creation of the most basic paradigm of heat transfer, the probability density function is not required to describe the motion; the operational definition of temperature via the BQ diathermal fiber [13] coupled to the Dhar-RLL condition Eq (30) for steady state systems is an adequate basis for the definition of temperature and the energetics associated with the temperature parameter. The description of the paradigm given here is independent of the results of future technical developments; the focus here is on the description of heat as given by various axioms and not on the mathematical and computational results that are based on various axioms that differ from what is being described here. Indeed, the survey of the field here shows that effort seems to be directed not so much to questions of interest to the natural philosophy of the area under investigation but more to the analytic and numerical solution of differential, integral and algebraic equations as things-in-themselves within fixed axiomatic structures that are not considered important for further consideration. For instance, it is acknowledged below by one prolific worker that despite the concept of temperature being ill-defined and controversial, nevertheless effort can be directed to novel mathematical outcomes—both analytic and numerical—irrespective of any precise definition of temperature. The work here, on the other hand does not have one iota of pure mathematical novelty—indeed the mathematics used here is trivial- but rather focuses on the basic concepts that have been used and continue to be used in framing equations and how it might be possible to re-conceptualize some of the basic propositions in thermodynamics, which could also influence the way mathematics is used to solve physical problems in thermodynamics.

### State functions, density functions and entropy considerations

**Fig 1:**
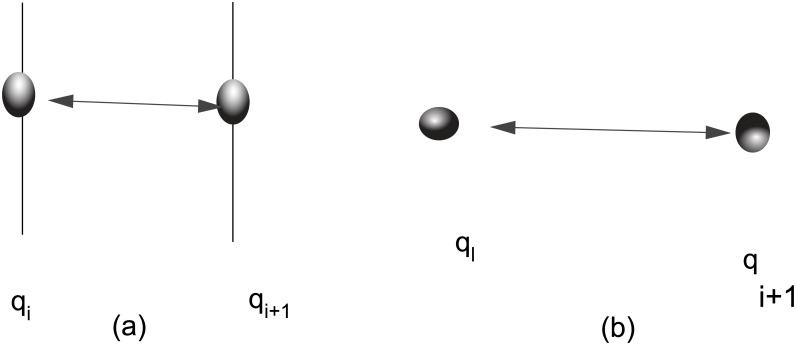
**Schematic (a)** represents adjacent particles that interact by hard sphere elastic collisions where a harmonic potential operates about the equilibrium position with no direct harmonic or other force coupling between these particles except for the hard sphere collisions. **Schematic (b)** represents a system where the adjacent particles interact continuously and directly though an inter-particle potential function, such as given in Eq (35).

The development of the Carnot cycle necessitated the construction of isothermal and adiabatic pathways within the cycle that were assumed to be mechanically “reversible” which lead eventually to the Kelvin-Clausius development of the entropy function *S* where for any reversible closed path C, $\oint_{C}dS=0$ based on an infinite number of concatenated Carnot engines that approximated the said path and where for each engine of infinitesimal extent Δ*Q*_1_/*T*_1_ + Δ*Q*_2_/*T*_2_ = 0 where the Δ*Q*’s and *T*’s are the heat absorption increments and temperature respectively with the subscripts indicating the isothermal paths (1, 2), (excerpt of E. Clapeyron, “Memoir on the Motive Power of Heat”, Fig 1, p.38 [18]) where for the Carnot engine, the heat absorption is for the diathermal (isothermal) paths of the cycle only.

Since “heat” has been defined as that form of energy that is transferred as a result of a temperature difference (p.73 [19], 1st cite, p.229 of [1, 20]) and a corollary of the Clausius statement of the Second law is that it is impossible for heat to be transferred from a cold to a hotter reservoir with no other effect on the environment, (p.10, Clausius deduction [5]) these statements suggested that the local mode of transfer of “heat” in the isothermal segments of the pathway does imply a Fourier heat conduction mechanism (to conform to the definition of ‘heat’) albeit of a “reversible” kind, but on the other hand, the Fourier mechanism is apparently irreversible, leading to an increase in entropy of the combined thermal reservoirs at either end of the material, leading at first sight to a paradox. These and several other considerations lead BQ [5, 13] to postulate that the Fourier heat conduction phenomenon to be another ancillary principle in thermodynamics, (p.9 [5]):

“We shall argue that Fourier’s theory contains a further thermodynamic principle … as a sort of addendum to it. This addendum we shall call … Fourier’s Principle, or simply **F**”.

The difference between the Zeroth law and **F** according to BQ is that the Zeroth law is meaningless for a single point, and for any two points it is indifferent to the mode of transport (conversion, radiation or convection). The Fourier principle (p.9, bottom par. [5]) is a local principle, which obtains for each point in the system if a temperature gradient is definable there. Here, we create a paradigm whereby the Second law, which according to BQ cannot be applied to **F** since the Second law is a global statement, actually implies the Second law where the energy transfer is that of one corresponding to optimized Carnot efficiency. This development then runs in the opposite direction to that envisaged by BQ, who could modestly claim that against their arguments, there might be a possibility of efforts that could result in subsuming Fourier conduction under the Second law (p.21, top par. [5]):

“The fact that contemporary engineering seems to have found no problems with its heat flow equations … confirms the reasonableness of taking Fourier’s inequality as a thermodynamic principle that can be sustained in its own right, unless perhaps, someday someone succeeds in subsuming it under the Second Principle”.

Whilst we maintain the truth of the inequality for strictly conductive heat transfer, with the principle being strictly local in nature, we do say that it it subsumed by the Second law, where the Second law statements can also be applied to this local process (in all cases within the limits of atomic dimensions). With hindsight, we understand that the gradient of the temperature requires the temperature to be defined at all neighborhoods of a point which does not obtain for atomic systems, and so a local effect would extend typically to a region of some atomic or particle diameters to suitably define gradients of thermodynamic variables; in the development here, it would extend at the minimum to the average distance between two particles. This work presents equations that model heat conduction as a thermodynamically reversible but mechanically irreversible process where due to the belief in mechanical time reversible symmetry, thermodynamical reversibility has been unfortunately and incorrectly linked to mechanical reversibility [8], that has discouraged such an association that is being made here. The modeling is based on an application of a “recoverable transition”, (Sec.3 Development of Kinetic Postulates, pp.168–170 [9], see esp. p.244 on new first order rate expression proposal [10]) defined and developed earlier on ideas derived from thermal desorption of particles from a surface where the Fourier heat conduction process is approximated as a series of such desorption processes, with due consideration for backscattering in the current formulation not made in former treatments. We recall that the original Carnot engine required both adiabatic and isothermal steps to complete the zero entropy cycle, and this construct lead to the consequent deduction that any Second law statement that refers to heat-work conversion processes are only globally relevant (p.11, bottom paragraph [5]). Here, on the other hand, we examine Fourier heat conduction as an essentially local process based on the results of MD simulations and model this process as a zero-entropy forward scattering process relative to each of the atoms in the lattice chain being treated as a system where the Carnot cycle can be applied individually. In order to deconvolve the various force interactions in this framework, and to ensure that both forward and backscattering processes are accounted for, recourse is taken to a simpler reference model which is generalized to the continuous-force lattice system of the MD simulations. The MD simulations illustrate that it is possible to give a quantitative interpretation to the theoretical constructs attempted here. Since the scope of the project is extensive, and where the concepts of temperature, equipartition and principle of local equilibrium (PLE) has not been extended to NE systems unambiguously, due to a lack of consensus, which did not discourage the development and application of mathematical and algebraic techniques to solve problems in the field. We limit ourselves to systems that do not seem to contradict these assumptions for NE systems, but the analysis here in not founded on these assumptions. We assume the validity of the PLE (p.7 [21]) but with augmented variables, unlike standard linear irreversible thermodynamics whereby for any steady state, the stationary thermodynamical variables corresponds to the equilibrium variables for the same quantity; in our case, we shall apply the assumption to the definition of temperature through the device of the BQ diathermal fiber to determine NE temperature [13], and relate the value determined by the diathermal fiber experimentally to the Dhar-RLL theoretical definition of temperature that obtains from an equipartition theorem; the energy variable from the First law obtains for all systems in any state. This PLE has been mathematically disproved [22] theoretically for non-augmented variables where the additional variables are required to described the NE state; subsequently, NEMD simulations of a hysteresis dimer chemical reaction subjected to extreme temperatures of several million Kelvins (and large temperature gradients of similar order) show that the principle breaks down [23–25], corroborating the previous theoretical result. NEMD simulations of a simpler non-chemical reaction of a LJ fluid system seemed to corroborate the validity of the Onsager reciprocity relations for extremely high temperatures and temperature gradients and results such as these gave support to using this principle for describing irreversible systems in general [26–28]. There is however a difference in how this principle is being used here, where two temperatures are associated with a particular particle; these temperatures are constructed relative to interactions at two different phases of the forward and reverse energy scattering processes. In complex interactions, simulations has shown that a virtual Gaussian (Maxwell-Boltzmann (MB)) energy distribution [29] associated with a fixed temperature may result as a summation of various NE processes with non-Gaussian or MB distributions. Hence there is no reason to assume that the energy profile over the “averaged” trajectory of the single particle would have a Boltzmann energy profile, but that the deconvolved backwards and forwards process could in the reference model. Indeed, in standard equilibrium applications, equipartition for instance applies only when the associated (**p**, **q**) momentum-spatial coordinates are “canonical” [30], a point often neglected in mathematical treatments. For this reason, only approximate theorems could be derived concerning energy-interconversion processes in the determination of reaction rates, [23–25, 31] which was ignored in some paralleling developments [27, 32]. Indeed in standard and stable renderings of the Fokker-Planck (FP) equation for systems characterized by a single temperature *T*, but where system particles are subjected to resistive forces, such as −*αv* with the resistive coefficient *α* coupled to the velocity *v* [33] in a random fluctuating force field *F*′(*t*) which arises from a Brownian heat bath for the particle of mass *m*, with *γ* = *α*/*m* the following dynamics

$$\begin{array}{c} m\frac{dv}{dt}=-\alpha v+F^{\prime }\left(t\right) \end{array}$$ (6)

leads to the second order, double moment Fokker-Planck expression for the velocity probability density *P* given an initial velocity *v*_0_

$$\begin{array}{c} P\left(v|v_{0}\right)={\left[\frac{m}{2\pi kT(1-e^{-2\gamma s})}\right]}^{1/2}\text{exp}\left[-\frac{m{\left(v-v_{0}e^{-\gamma s}\right)}^{2}}{2kT(1-e^{-2\gamma s})}\right] \end{array}$$ (7)

where *s* is the time elapsed. What is interesting in Eq (7) is that a temperature *T* can be associated with a NE profile, which transforms to the time-independent profile when *s* → ∞ for that specified temperature *T*: Eq (7) is useful for transient isothermal phenomena, where a zero current steady state obtains when *s* → ∞ and may be contrasted to the non-zero steady state current probability of Attard for instance (Eq 63 [34]) where over a smaller subsystem length *L* (theoretically, the limit *L* → 0 exists) whose ends are at ±*L*/2 and are maintained at *T*_+_, *T*_−_ respectively. Making use of various assumptions, such as the adiabatic evolution of the Hamiltonian (meaning no heat flow to the isolated system), and the cardinal time-reversibility property (Eq 4 [34]), a steady state probability density (Eq 63 [34]), supposedly non-“Gaussian” is derived consisting of exponential terms in the numerator and a type of steady state partition function in the denominator. The connection of this and other related work to the Jarzynski (J) equality is noted (p.224103–8 [34]); J is discussed in later sections. The work here, on the other hand is focused on “local” properties (within limits of molecular distances). In response to the above, we conjecture that the equilibrium density is a special case of the NE constant temperature steady state with non-zero independent fluxes and forces (**J**,**X**) respectively, where the same algebra can be used in deriving the density distributions for the various ensembles (Chap.2 [35]). Thus if the system Hamiltonian operator is $\hat{H}\left(\hat{p},\hat{q};\Omega \right)$ where (**Ω** ≡ (*V*, *N*)) for the equilibrium state, then solving the quantum Schrodinger equation ${\hat{H}}_{\Omega }\Psi_{\Omega ,i}=E_{i}\Psi_{\Omega ,i}$ leads to the energy eigenvalue spectrum *E*_*i*_ from which the density is derived by standard methodology (Chap.2 [35]). Solving for the steady state condition ${\hat{H}}_{\Omega '}^{}\Psi_{\Omega^{\prime },i}=E_{i}\Psi_{\Omega^{\prime },i}$ where (**Ω**′ = {**Ω**^**′**^ ∪ (**J**,**X**)} will according to the conjecture provide a local density distribution with the temperature determined by the BQ diathermal fiber. Relative to the ${\hat{H}}_{\Omega }$ equilibrium Hamiltonian, the density *ρ*_**Ω**^**′**^_ will have a structure of a possibly non-Gaussian perturbation. We show (paragraph after Eq (45)) that our theory of recoverable transitions is *not*(a) dependent on the shape of the probability distribution and its departures from the thermostatic quantities nor (b)on equipartition results that refer to the potential energy. The generalized temperature is determined by the Dhar-RLL result Eq (45).

BQ have extended the operational definition of NE temperature by recourse to the “diathermal fiber” [5, 13] which extends the Zeroth law to NE systems; if there is no net flow of energy between regions (quasi-points) *A* and *B* that is connected by this fiber, which can only conduct thermal energy with no transport of matter, then these regions are defined to be at the same temperature. Implied in this definition is a connection to the thermostatic systems. Thus if our reference system is known to be in thermostatic equilibrium, (say located in region *B*) with temperature *T*, and the NE system is at region *A*, with a designated temperature $\hat{T}$, then according to this definition $T=\hat{T}$. The definition allows one to deconvolve a complex NE process into one that is composed of different temperatures that are equivalent to the thermostatic temperatures ascribed to the same region for these different processes, for instance we show different temperatures obtaining for the forward and back-scattering processes in our lattice chain. From above, we note that the density-in-phase distribution is either rescaled in the temperature as in the FP example above, or else the density-in-phase distribution is anomalous [34], so that the equipartition results that are obtained from averaging using these distributions will not provide the standard thermostatic results even for the same local Hamiltonian, since there is ambiguity with regard to the temperature or the density-in-phase distribution used in averaging. In this work, the density distribution is of no relevance; the NE temperature will be determined by recourse to the BQ fiber and the Dhar-RLL result of Eq (45). On the other hand if a deconvolution is attempted, then it becomes possible to define NE temperature obtaining for each of the separate processes subjected to the axioms of the Second law obtaining for these systems; the first axiom used here is that for each of these separated processes, the temperature would be the same as for that determined by the diathermal fiber. However, since we are partitioning the processes, the time coordinate for a sequence belonging to a particular process is discontinuous in some neighborhood of that variable. It is not obvious how this partitioning scheme is equivalent to the methods currently used, where there is a conflation of these various processes [34, 36–39]. The interest of researchers, judging from the literature, are the dynamics of processes as described by the solution of differential, integral and algebraic equations that are set up with different boundary conditions; the content of the research being primarily the ability to solve and describe these solutions and the reasons why these equations were selected. The interests here, however, focuses on the relationship of heat transfer and the Second law, and the consequences of those relationships using the primary definitions provided by the pioneers of thermodynamics over the last few centuries.

The equations that derive from the paradigm developed here shows that the “work” done in the transfer of energy in the single particle representation are at optimal Carnot efficiency—which is the conductive energy due to a temperature difference—and is equal in magnitude to the energy transfer rate due to Fourier conduction, which by definition is the heat transfer rate. This implies that the two forms of energy are not mutually exclusive, as assumed in standard thermodynamic analyses, and that a more generalized treatment that can subsume both these forms of energy in thermodynamics is feasible. Such a detailed exploration is reserved for future consideration. The MD simulations show that it is possible to give a numerical interpretation to each of the constructs attempted here. Such views and results as these, if developed to a successful conclusion could imply that the Carnot cycle can be viewed as describing a local process of energy-work conversion and that irreversible local processes might be brought within the scope of this cycle, implying a unified treatment of irreversible, reversible, isothermal and adiabatic thermodynamic processes.

Principles were developed in connection with the thermal desorption problem, where it was proven that the irreversible desorption of particles on a surface [9] within a relaxation time *τ* conforms to an adiabatic scattering zero entropy process along the center-of-mass frame of the desorbing particles (Sec.4.4, Eqs (85–98) and Eqs (30–44)resp. [9, 10]). To summarize the more detailed treatment found in (Sec. 4.4 [9]), for transitions between states *i* with probability *p*_*i*_ before a transition and $p_{i}^{\prime }$ after, where state i transmutes into another state (denoted by primes), the entropy change Δ*S* assuming the form given by equilibrium thermodynamics and assuming the PLE is

$$\begin{array}{c} \Delta S=kk^{\prime }\Delta \sum_{i=1}^{N}p_{i}\text{ln}\;p_{i}=0 \end{array}$$ (8)

for the entire transition of all states *i* imply

$$\begin{array}{c} p_{i}\left(E_{i},T\right)=p_{i}^{\prime }\left(E_{i}^{\prime },T^{\prime }\right). \end{array}$$ (9)

A recoverable transition is defined as one that conserves the probability of an energy channel or microstate of the system such that Δ*S* = 0. This condition is met whenever *S* = *S*(*P*), for probability distribution *P*, where *δP* = 0. The above follows from the fact that the events associated with process with energy state *i*′ follows directly from state *i*, conserving the probabilities [8, 9, 17]. This leads in turn for any of the canonical Maxwell-Boltzmann, Fermi and Bose-Einstein probability distribution to the relation

$$\begin{array}{c} E_{i}/\left(kT\right)=E_{i}^{\prime }/\left(kT^{\prime }\right) \end{array}$$ (10)

where the *E*’s are energy variables between the two states with defined temperatures *T*, *T*′ and where the partition function Z too is conserved, i.e. $Z=Z^{\prime }$ (Eq 65 [9]), and where if we summed the energies in Eq (10), we have the heat energy $Q=\sum_{i}^{n_{t}}E_{i}$ conforming to (Eq 73 [9])

$$\begin{array}{c} \frac{Q}{kT}-\frac{Q^{\prime }}{kT^{\prime }}=0 \end{array}$$ (11)

or the entropy is conserved for this “heat pulse” movement which has been described as a recoverable trajectory. In this theory, the *Q*’s represent the total energy of the ensemble of particles (Sec. 3–4, pp.168–179 [9]). We note that the prime and unprimed variables refer to the same system making a transition. These transitions are “thermodynamically reversible” in the sense of zero entropy change [8], but is clearly mechanically irreversible [8]. The above system follows [9, 10] an event streamline, where each event is separated by a relaxation time *tau* leading to the primed state; clearly this transition is non-cyclical, and the initial and final states differ in spatial location as well as in the thermodynamic state;. For the *ith* state transiting a sequence of states **q**, the probability does not change (Eq 14 [9]) i.e. $P_{i}^{q}=c_{i}$ or $\delta P_{i}^{q}=0$ as in the thermal desorption problem [10], which is a mechanically time irreversible process leading to the disintegration of the system as particles leave the surface. It was previously proved that the concept of time-reversal invariance, a central tenant in the description of physical systems is a mathematical contradiction [7, 8, 17, 40] in the manner that it is applied mathematically to physical models of systems. Nevertheless, research still persists that depicts this natural irreversibility or absence of time-reversal invariance as an anomaly leading to discovery of high impact [41]. If the NE entropy S is written as S=S(Σ,Ω,P(t)) per particle of the disintegrating ensemble, where *Σ*, *Ω*,**P** and *t* are the NE, thermostatic, probability spectrum and time variables respectively, and for any fixed (*Σ*, *Ω*) = Λ then the recoverability condition *δ* **P** = 0 in the NE domain within a relaxation time *δt* = *τ* implies δS(Λ,P(t))=0. If all the particles on a surface are at temperature *T*_*b*_, then the particle flux after desorption is *T*_*a*_, then *T*_*b*_ > *Ta* (p.178, ref. 40 within of first citation [9, 42]), the total work function ${\bar{W}}_{c}^{+}$ which includes the kinetic energy of the center of mass velocity of the particle ensemble is such that δS=0 and ${\bar{W}}_{c}^{+}=<Q_{b}>-<Q_{a}>$ fulfilling the conditions of a recoverable trajectory (Eqs 97–98 [9]). For the usual first order kinetics desorption problem, the rate constant *k*_*d*_ based on this model of recoverability theory was later shown to be (Eq (44) [10])

$$\begin{array}{c} k_{d}=2{\left.\frac{dG(\bar{X}/\left(\left(R\left(0\right)\left(1-a^{2}\right)\right)\right)}{dt}\right|}_{\tau \to 0} \end{array}$$ (12)

where *R*(*τ*) is the autocorrelation function for white noise (Gaussian temperature heat bath of the surface)

$$\begin{array}{c} R\left(\tau \right)=\frac{kT}{\kappa (m)}\text{exp}-\alpha |\tau |(\cos\beta \tau +\frac{\alpha }{\beta }\sin\beta |\tau |) \end{array}$$

where $\bar{X}=\sqrt{\frac{2\bar{W}}{\kappa }}$ is determined from experiment and the error function is

$$\begin{array}{c} G\left(X\right)=\frac{1}{\sqrt{2\pi }}\int_{-\infty }^{X}\left(\text{exp}\frac{-y^{2}}{2}\right)dy. \end{array}$$

From the *Theorem* (p.177 [9]) this disintegrating system coupled to a definite temperature reservoir for the surface is frictionless. In particular, it is mechanically irreversible since the particles cannot traverse past the surface, and the intermolecular potential allows only for escape through the forward motion during molecular desorption.

For the above system, [10] that is disintegrating, the ad-atoms on the surface of the substrate eventually all leave the surface, and each of these ad-atoms have instantaneous energy *E*′, where $E^{\prime }=E-\delta W$, δW being the work done by the particle on the force-field it traverses, which is absorbed into the field. The particles then move on to infinity in space if no work potentials are present along the trajectory when the kinetic energy of the particles are still positive. Clearly for this situation, no structure is preserved of the original system, and there is unidirectional motion. Of interest then is to investigate the possibility of modeling energy transfer processes that has something of the form above of zero entropy trajectories relative to the defined heat terms, but where the system is not disintegrating, i.e. where structural integrity is preserved as in typical descriptions of thermophysical systems in the steady state. The reason why this system is relevant is because of the temperature gradient, i.e. the temperature decreases along the streamline, which is consonant with that of conductive heat transfer. However, a critical issue here is to ensure that no violation of the Second law is implied in framing the model. Thus some Second law consideration is warranted to ensure no violation. The Clausius statement C of the Second law (p.184 [43]) may be stated as:

“No process is possible whose sole result is the transfer of heat from a cooler to a hotter body”

whereas the Kelvin-Planck K version (p.185 [43]) is

“No process is possible whose sole result is the absorption of heat from a reservoir and the conversion of this heat into work”.

The subtle proof of their equivalence is well-established (p.185–187 [43]). Thus a refutation of K refutes C, and so experiments or rather simulations that apparently contradicts C cannot be categorized as heat transfer according to **F** as framed by BQ. Therefore the conductive heat system that is chosen in what follows must locally on average not contradict *C*. Therefore, **F** provides a criterion to distinguish, at the local level, whether or not heat conduction is taking place. Several inferences of a faulty nature has been pointed out (p.179, Sec. 5 [9]) with regard to the Feynman rachet and pawl device (where no proof was given to show violation of the Second law in the original Feynman exposition (Chap.46 [44]) which was used as a basis to derive microscopic laws from the macroscopic, with apparent endorsement of Prigogine and in line with his philosophies [45]. The reference to this device—which is *not disintegrating*—to derive microscopic laws suggests the possibility of deriving work from a single temperature source in violation to the K principle. More recent works has in fact argued very strongly for the impossibility of such a device existing in nature e.g. [46], thereby proving as a consequence that C is a true proposition due to the C-K equivalence. It can be inferred from this fact alone that the Kondepudi analysis is not tenable, in addition to all the other detailed arguments of non-tenability [9]. Thus systems analyzed theoretically, or in experiments and simulations must be carefully constructed to have the minimal number of variables so that no contradiction of C or K ensues [47]. An incomplete set of variables is at the heart for instance of the Maxwell analysis under the section “Limitations under the Second law” (Chap.22, p.338 [48]). A more complete description of the Maxwell demon using quantum mechanics given by Brilloun [49] resolved the issue when he took into account the photons that were required to probe the particles that had to require energies satisfying the inequality (Eq (4) [49]) *hν*_1_ > >*kT*_*o*_, where the blackbody radiation of the cavity was an energy masking factor that lead to the anomaly reported by Maxwell. We note that the current arguments refer to mechanical systems without coupling to the electromagnetic fields, where the arguments above are not complete. We shall not refer to such coupled systems in this work concerning the Zeroth law and temperature differentials that might be indicated at a theoretical [50] or experimental level [51] in this work. Thus systems that contradict C cannot be considered as heat conductive transfer in the BQ sense at the microscopic level whenever there is violation of **F**. This means that the system described in [52] with alternating masses in an FPU lattice chain cannot locally be considered as pertaining to Fourier heat conduction in the BQ sense or the C statement. This implies that the theoretical analysis must be such that the minimum size of the system must be such that **F** always holds between two contiguous regions; in an atomic system, the temperature gradient between two neighboring particles must have a temperature gradient that satisfies **F**. This criterion then implies that further variables, as in the Brilloun analysis of the Maxwell demon must be used to show work- heat conversion taking place between contiguous regions. In order to conform to the Fourier principle strictly, all energy conducting phenomena due solely to a temperature difference must be free of conversion processes. Interestingly, in the inhomogeneous lattice chain model, [52] it is claimed that the steady state temperature profile is “saw tooth” in nature such that positive gradients exists in alternate segments along the direction of heat flow, in contradiction to C. For such a system of alternating particle mass magnitude of ratio 2.62, the lighter particles are hotter than the adjacent heavier ones such that there is a periodic sign change of the gradient value and “it is not because of a thermal conductivity *K* that oscillates on the microscopic scale” (p.184301–2 [52]). If this conductivity argument is correct, then the **F** principle **q**.∇*T* ≤ 0 is violated locally, if we presume that the heat current energy transfer is from the hotter to the colder reservoir, and that this same nonzero direction of transfer obtains for all neighboring regions where the principle is violated. If we consider the portions to the left and right of a segment consisting of the two unequal masses coupled by force fields as representing homogeneous reservoirs with fixed temperatures corresponding to the temperature of the mass it is coupled to in the segment, for each of the reservoirs, then the Carathéodory definition of heat and C statements of the Second law would be violated if the energy transfer referred to heat with no work inter-conversion, in addition to **F** for adjacent segments *i*, *i* + 1 respectively, where *T*_*i*+1_ − *T*_*i*_ ≥ 0 if the average energy flux across the masses are on average the same as when *T*_*i*+1_ − *T*_*i*_≤0, which follows from energy conservation. The apparent paradox(es) may be resolved if it is understood that the energy flows that result from force interactions between dissimilar particles (an analogy here is the Peltier effect in thermoelectrics where the coupling of an electric current along dissimilar material interfaces leads to a reversible heat absorption) involves work-heat conversion and therefore (a) cannot be viewed as a local phenomenon in the BQ sense and (b) is not conductive heat in the BQ, Clausius and Carathéodory sense in order for the Second law to hold; the Second law was conceptualized within the framework of matter existing as a continuum if no vacuum were present; the atomic paradigm used here implies that these statements must be qualified so as to allow for both the continuum and atomic paradigms, where an appropriate averaging process over some atomic dimensions defines a spatial point in the continuum model. Thus for example, one might extend the Clausius Second law statement $C_{e}$ to:

“No process is possible whose sole result is the transfer of heat from a cooler to a hotter body through a locally homogeneous medium”

where a locally homogeneous medium is defined as one in which the particle masses and/or force fields are exactly the same over all the equivalent lattice points of the periodic array or grid to cater for the possibilities suggested by some investigators [52, 53] mentioned above. With such a definition, many of the conductive heat systems studied would not be defined as such at the atomic level where thermal rectification and other effects due possibly to phase transitions occur [53–64]. Hence it is essential to reduce a heat conduction system to its simplest possible form that is consonant with $C,C_{e}$ and **F** for studies that wish to relate to all these stated principles. On the other hand, the system must be completely described, so that no contradictions arise with regard to the Second law, as in the case of systems that admits a Maxwell demon or its equivalent ([45, 47, 49, 65], chap.22, p.338 [48]) because of incomplete description. The criterion used here is that if the system description and dynamics based on a paradigm does not contradict the Second law, mechanics and energy conservation, then it is an admissible theory that may be compared and contrasted to other paradigms and viewpoints that fulfill the same criterion.

### Comment of some relevant current trends in heat conduction and other energy transport problems

In as far as the elementary recoverability transition is defined as the equality *δS* = 0, it must be discussed in relation to other developments involving equalities. It was explicitly stated that the Liouville (**p**, **q**) space also used in the Hamiltonian development was *not* the space considered (p.169, top par. [9]) in the trajectory analysis, and this was also reiterated in the development of a stochastic equation to replace the Liouville equation in [66, 67] due to its general non-validity. For reactive systems, such as applied to [68, 69] a macromolecular (chemical reaction) system NE transition

$$\begin{array}{c} \text{A}⇋\text{B} \end{array}$$ (13)

the J concept [70–72] initially utilized equilibrium statistical mechanical expressions (e.g. Δ*F* = −*β*^−1^ ln(*Z*_1_/*Z*_0_)) for free energy difference variable Δ*F* and partition functions *Z* with *β* = 1/(*kT*) to derive

$$\begin{array}{c} \bar{\text{exp}(-\beta W)}=\text{exp}\left(-\beta \Delta F\right) \end{array}$$

where supporting and complementary variants have been further adduced e.g. [73] using master the equation approach that assumes time reversibility -shown to be untenable [8]; J assumes also the Liouville equation in a deduction (Eq (9) [8]). Hänggi and Talkner [74] also describe more recent elaborations of this equality assuming time-reversibility; a common theme in this development is the use of a transition Hamiltonian $H_{\lambda }\left(z\right)$ described in **z**(*λ*(*t*)) = (**p**, **q**) space thereby implying that canonical coordinates are being utilized [30], and where the PLE is assumed with thermostatic potentials of quantum statistical mechanics such as the free energy *F*_*λ*_ = −*β*^−1^ ln*Z*_*λ*_ at all times during the transition described by the parameter *λ*(*t*). A Hamiltonian that is explicitly time dependent is not generally a constant of the energy and furthermore if it is axiomatically defined to be the energy content of the system under consideration, then an elaborate yet to be proven theorem is required to show that under canonical coordinate transitions, it is possible under constant temperature conditions at the very least, a general path must always exist such that the transition along this coordinate frame involves *only* work transition with no reference to heat when both quantities are represented in the Hamiltonian that is axiomatically taken to represent the energy U in all of mechanics and statistical mechanical theory where <H>=U=W+Q where W and Q are the work and heat energy content relative to a defined standard state. Nevertheless, assume for the moment that an adiabatic pathway exists, where the Hamiltonian transits in such a manner that

$$\begin{array}{c} W=\int_{0}^{t_{s}}dt\dot{\lambda }\frac{\partial H_{\lambda }}{\partial \lambda }\left(z\left(t\right)\right). \end{array}$$ (14)

so that instead of the inequality (Eq (1) [70]) $\bar{W}⩾\Delta F$ one can calculate

$$\begin{array}{c} \bar{\text{exp}(-\beta W)}=\text{exp}\left(-\beta \Delta F\right) \end{array}$$

where

$$\begin{array}{c} \Delta F=\int_{0}^{1}d\lambda \left\langle \frac{\partial H_{\lambda }}{\partial \lambda }\right\rangle . \end{array}$$ (15)

From a paraphrased Carathéodory statement of the Second law (pp.198–206 [43]): “In the neighborhood of any equilibrium state of a system characterized by an arbitrary number of variables, there exists states that are not accessible by a reversible adiabatic pathway” lead to a theory where for the above system described by the First law differential

$$\begin{array}{c} dQ=dU+\sum_{i=1}^{f}Y_{i}dX_{i}=dU+dW \end{array}$$ (16)

with the *Y*’s representing forces, *dW* the increment of work done by the system on the environment, the *X*’s the conjugate extensive variable of the forces, *U* the system energy with heat content *Q*, there exists an infinite number of non-intersecting surfaces described by the same function *σ*_*X*_(*U*,**X**) where each non-intersecting surface *i* is given by a real number *c*_*i*_ so that the surface is described by *σ*_*X*_(*U*,**X**) = *c*_*i*_; instead of the energy, one can also use the temperature variable **t** so that for each *c*_*i*_, the equation *σ*_**t**_(**t**,**X**) = *c*_*i*_ defines an adiabatic state uniquely. Since **X** may be taken to be independent over a certain domain D, the fact that two interrelated variables are used to characterize the surface implies that the inverted equation **t** = *g*_**t**_(*c*_*i*_,**X**)≠*C* where *C* is a constant for all **t**,**X** where certain conditions of the implicit function theorem concerning the determinant of the differential matrix *g*_**t**_ must be met for this routine condition to hold [75]; for this situation, **t**_1_ ≠ **t**_2_, where *X*_1_ ≠ *X*_2_ for some $X_{1},X_{2}\in D$. As pointed out below, the free energy differential $dF=-SdT-\sum_{i}^{m}\chi_{i}dx_{i}+\sum_{j}\mu_{j}dN_{j}$ refers to the total adiabatic work done whenever −*SdT* = 0 for the canonical ensemble where there is no need to consider the chemical potentials for *δN*_*j*_ = 0; from Carathéodory’s theory, we have in fact *dQ* = *λdσ*_**t**_. Then from Eqs (16, 15), with *dQ* = 0, and the fact that the Hamiltonian transitions are taken to be ensemble averages over the end points, we can write

$$\begin{array}{c} dQ=0=\int_{X_{1}=F_{c}\left(\lambda =0\right)}^{X_{2}=F_{c}(\lambda =1}d\lambda \left\langle \frac{\partial H_{\lambda }}{\partial \lambda }\right\rangle +\sum_{i=1}^{f}Y_{i}dX_{i} \end{array}$$ (17)

where *F*_*c*_ is a mapping function from *λ* to the **X** domain, leading to

$$\begin{array}{c} -\Delta W{\left.\right|}_{X_{1}}^{X_{2}}=\int_{0}^{1}d\lambda \left\langle \frac{\partial H_{\lambda }}{\partial \lambda }\right\rangle \end{array}$$ (18)

but where in general, the temperatures differ during the transition since here *t*_1_ ≠ *t*_2_ for a specific class of adiabatic transitions that can be equated with work-only terms for the canonical system. Generally of course,

$$\begin{array}{c} \int_{X_{1}}^{X_{2}}d\lambda \left\langle \frac{\partial H_{\lambda }}{\partial \lambda }\right\rangle =\Delta W+\Delta Q\neq W \end{array}$$

where the endpoints are at *X*_1_, *X*_2_ corresponding to *λ* = 0, 1. However, assuming this is the case for all transitions, where the Hamiltonian setup refers to work only changes between any point *X*_1_ and *X*_2_, then the temperatures at the endpoints of the transition is not constant in general, contradicting the defined conditions of the canonical ensemble used to derive the J equality; all systems using such results refer to constant temperature conditions during the transitions (Eqs 2(a, b), 5, 7 etc. [70]). The only other possibility given that the equality is correct is that it is true only over a certain class of pathways, with a continuously changing Hamiltonian, but the functional forms have yet to be specified, at least in detail. We note that in (par. before Eq (9) [70]), canonical coordinates [30] are specified (i.e.**y** = (**z**,**z**′)). Such assumptions are commonplace [76], together with the fluctuation-dissipation theorem that depends on on time reversibility and its inference that the NE entropy has a form that connects densities *P* (Eq (1) [77]) such that $P\left(\Delta S\right)/P\left(-\Delta S\right)=e^{\Delta S/k_{B}}$, and from this the curious result for non-disintegrating systems < *P*(Δ*S*)/*P*(−Δ*S*) >= 1 results in $<e^{-\Delta S/k_{B}}>=1$ (Eq (2) [77]). Clearly, the entropy still has to be defined from thermostatics, and changes to *S* are attributed to the result of the NE fluctuation-dissipation theorem; Kim for instance has stated “the symmetric fluctuation associated with forward and backward manipulation of the NE work is contingent on time-reversal invariance of the underlying mesoscopic dynamics” which would lead to the ratio of the densities above to be unity [78]. In addition, the Kondepudi zero entropy analysis was shown to be flawed (Sec.5.1 [9]), since it refers in its algebraic structure to the Feynman rachet and pawl type devices which was later shown to be untenable, [46], we do not here develop any reference to the condition < *P*(Δ*S*)/*P*(−Δ*S*) >= 1 in our irreversible condition *δS* = 0, since in the development of our paradigm below, mechanically irreversible back-scattering processes are implied, in addition to a temperature shift, which is *not* featured at all in the J development and its derivatives [70–72, 77, 79]. It is noted that in recoverability theory, the condition of the probability of states *δ***P** = 0 (conservation of probability for events *P*_*i*_)when applied to systems exhibiting such transitions, and using the equilibrium form of the partition function $Z_{R}$—as an approximation, as with J and subsequent developments—leads to the identity (last par., Sec. 4.3, p.177 [9]) $\text{ln}(Z_{R}/Z_{R}^{\prime })=0$ due to the simultaneous change in both temperature and energy levels, a situation not contemplated in the J development where indeed the above condition for the isothermal system obtains when (Eq (8) [70]) < exp(−*βW*) >= *Z*_1_/*Z*_0_ = 1 or for the trivial case when the average ensemble work is zero. Most treated cases, as in chemical transformations given in Eq (13) and discussed for instance in [68] are for isothermal non-zero work transitions.

Further, the fact that the Helmholtz free energy expression was used implies a canonical distribution with the exponential term that sets the partition function in the denominator as a normalizing factor; some e.g. Attard [34] presented a development where the non-equilibrium density is not of the simplistic form used in [70]; however, time reversibility is a centerpiece of this development which has a sequel, some highlights therein include [36–39]. We note that the J development definitely utilizes the Helmholtz free energy *F* (par. after Eq (8) [70])

$$\begin{array}{c} \Delta F=-\beta^{-1}\text{ln}\left(Z_{1}/Z_{0}\right) \end{array}$$ (19)

with differential (Eq (8.7.10) [33]) *dF* = −*SdT* − *pdV* + ∑_*j*_ *μ*_*j*_ *dN*_*j*_ or in general for *m* force terms *χ*_*i*_, $dF=-SdT-\sum_{i}^{m}\chi_{i}dx_{i}+\sum_{j}\mu_{j}dN_{j}$ which interestingly implies that the Helmholtz energy at constant temperature would yield the increment of the total work done by the system *without* heat transfer, since by definition the heat increment is *dQ* = *TdS* in standard notation; the simplified form of the Gibbs free energy on the other hand would have the expression $G=-\beta^{-1}\text{ln}\left(Z\right)+PV=\beta^{-1}\left(V{\left(\frac{\partial \text{ln}Q}{\partial VB}\right)}_{N,T}-\text{ln}Z\right)$; (unfortunately), experimentalists have occasionally identified the Helmholtz with the Gibbs energy [80] in such chemical transformations in order to determine the approximate work increments. These and the ambiguity of the theory, not least the use of thermostatic potentials for far from equilibrium processes and the nature of the work involved could very well mean that the theory is approximately correct most of the time, but that some systems have energy distributions that creates measured discrepancies between theory, simulation and experiments [69]: for instance the discrepancies found in the K^+^ ion in the gramicidin channel could well be due to the non-canonical coordinates used that in that particular case leads to a severe distortion of the probability density distribution due to the approximate nature of the theory, in addition to the ambiguity of relating the Hamiltonian to an apparent work only transition.

There were at least 3 different ways in which NE principles and properties were deduced in previous studies [3, 6, 9, 81, 82]. Firstly, if the PLE is assumed as a strong principle [26, 28, 76], then thermostatic potentials such as given by [70] for NE systems and using time comparisons for irreversible and reversible transitions (Eq 33 [3]) e.g.

$$\begin{array}{c} \int_{B}^{A}\frac{\partial S^{ret}}{\partial t}dt=\int_{B}^{A}\frac{\partial S^{irr}}{\partial t}dt \end{array}$$ (20)

reminiscent of [70], where a continuous form of the Liouville equation is used (par. between Eq (7) and Eq (8) [70]), where with independence of switching times, (Eq (9–10) [70]) it is concluded that

$$\begin{array}{c} \bar{\text{exp}-\beta W}=\text{exp}(-\beta \Delta F); \end{array}$$

Eq (20) with fixed time transition endpoints on the other hand lead to an inequality (Eq (46) [3])

$$\begin{array}{c} \gamma_{A}\left(t\right)=\sigma_{irr}\left(A\right)-\sigma_{ret}\left(A\right)\geq 0 \end{array}$$ (21)

which is a general principle of minimal entropy production which subsumes Prigogine’s entropy production principle which obtains under a restricted set of conditions (Sec. 12.B.1 [83]). Comparison of transition times in this case leads to an inequality.

Secondly, if circular integrals are taken over the thermodynamical space of a steady state system for energy U and entropy S respectively (Eq (1–2) [6]), then exact coupling relations are derived, such as a new result for the Kelvin heat engine (Eq (43) [6]). These equalities refer to a canonical distribution that admits of both heat and work increments for a subsystem within the system, which is not evident in [70] from the discussion given above.

Thirdly, in non-synthetic MD studies involving hysteresis chemical reactions [24, 82], it was shown that the probability density for non-canonical coordinates, such as obtains along a chemical reaction trajectory from reactant to product and vice-versa was in general non-Boltzmann by direct sampling. This has important implications for computing standard transitions between species that assumes the J equality, when the probabilty density is non-Boltzmann therefore implying that the form of the work energy as calculated directly from the Helmholtz free energy expression for a standard Hamiltonian in the canonical ensemble Eq (19) from the partition function is not exact. For a total system Hamiltonian *H* that models a chemical reaction written

$$\begin{array}{c} H\left(p,q\right)=\sum_{i=1}^{n}\frac{p_{i}^{2}}{2m}+\sum_{i<j}V\left(q_{i}-q_{j}\right) \end{array}$$ (22)

the method of statistical mechanics leads to the probability distribution having the form *ρ*(**p**,**q**)∼exp[−*H*(**p**,**q**)/(*kT*)] and so for the separable Hamiltonian coordinates, the kinetic energy $E_{k,i}=\frac{p_{i}^{2}}{2m}$ and potential form *V*(|*r*_*i*_ − *r*_*j*_|) would have Boltzmann distributions. The internal coordinates for artificial aggregations such as molecules are characterized by relative velocities and positions for any two particles *k*, *l* forming a molecule at a certain time interval, where one could write for instance $P_{j}=p_{k}+p_{l}\;,\;R_{j}=\frac{1}{m_{k}+m_{l}}\left(r_{k}+r_{l}\right),\;\;k\neq l$. Permanent aggregated states may be expressed as canonical transformations **Q** = **Q**(**p**,**q**),**P**(**p**,**q**) [30] and the Hamiltonian in the transformed coordinates must in a canonical ensemble also have the Boltzmann density distribution; for systems using internal coordinates that are not canonical, no general theory exists that can predict even for equilibrium systems the density profile; nevertheless theories have been created that assumes Boltzmann densities for these internal coordinates [32, 76]. The intermolecular coordinates (**r**_*i*_,**r**_*j*_) about a bond of a molecule that is both forming and breaking, is relevant to depict the internal kinetic energy of the bond $K_{int,i,j}=\frac{1}{2}\mu {\left(v_{i}-v_{j}\right)}^{2}=\frac{1}{2}\mu {\left(\dot{r}\right)}^{2}$ termed IKE and the total internal energy of the interparticle pair TIEC is given by $E_{mol,tot}=\frac{1}{2}\mu {\left(\dot{r}\right)}^{2}+V(|r_{i}-r_{j}\left|\right)$. Detailed equilibrium MD simulations show that for the hysteresis chemical reaction considered [31], IKE shows a steady state density that does not have a Boltzmann profile (Fig 4, p.896 [24]); TIEC too does not feature a Boltzmann density profile (Fig 5, p.897 [24]). The very formidable literature that has accompanied the J, Crookes and allied equality developments that uses the statistical potentials that presupposes the quantum Boltzmann density profile could therefore be expected to be generally approximate in nature [68, 69, 72, 74, 74, 80]. For instance, In the case of the dimer dissociation reaction

$$\begin{array}{c} 2\text{A}⇌{\text{A}}_{2} \end{array}$$ (23)

the absence of an accurate probability density meant that (Eq (33–34) [82]) only an approximate expression was written in the absence of the exact knowledge of the density function e.g.

$$\begin{array}{c} T\left\langle \Delta S\right\rangle =T\int_{r=0}^{r_{f}}\Delta S_{r_{f}}^{r_{2}}P\left(r_{2}\right)dr_{2}\approx \bar{W_{rf}} \end{array}$$ (24)

or

$$\begin{array}{c} \left\langle \Delta S\right\rangle \approx \left(\frac{\bar{W_{rf}}}{T}\right) \end{array}$$ (25)

where $\bar{W_{rf}}=\Delta G_{mol}$ (Theorem 3, Eq 32 [82]) is the total change in free energy about the bond trajectory. Exact forms require a steady state density profile, such as (**Theorem** 5, Eq (64), p.225 [82]) the standard enthalpy of reaction $\Delta H^{0–}\left(T\right)$ is given by

$$\begin{array}{ccc} \Delta H^{0–}\;\left(T\right) & = & \xi_{max}+\int_{r=0}^{r_{b}}\left(\Delta W_{r_{f}}^{r_{2}}\left\{P\left(r_{2},T\right)-T\frac{\partial P(r_{2},T)}{\partial T}\right\}\right.\\ & - & \left.P\left(r_{2},T\right)T\frac{\partial \Delta W_{r_{f}}^{r_{2}}}{\partial T}\right)dr_{2} \end{array}$$

where for instance the probability density is given by *P*(*r*_2_, *T*). The exact expression for the entropy expression given above Eq (25) becomes (Eq (62),**Theorem** 4, p.224 [82])

$$\begin{array}{c} \Delta S^{0–}\;\left(T\right)=-\int_{r=0}^{r_{b}}\frac{\partial }{\partial T}\left(\Delta W_{r_{f}}^{r_{2}}P\left(r_{2},T\right)\right)dr_{2}. \end{array}$$

Prominent workers in non-equilibrium thermodynamics theory do not feature the density factor in their foundational work [27, 32, 76].

Nevertheless, it is essential to relate the current developments to the extensive technical literature that seeks to illustrate and explain phenomena by solving differential, integral and algebraic equations associated with models not focused on the axiomatics mentioned here, **F**, and the Second law, nor on the qualifications to them made here. Most other works cited here write down a conservative Hamiltonian subjected to gradients of temperature to illustrate various phenomena. One interesting development does away with the Hamiltonian description since it is opined that most NE systems do not converge to an equilibrium state that is typical of Hamiltonian systems (Sec.1., p.032131–1 [59]); in particular, very little focus is given to the detailed balance condition that is used in the updating procedure for Monte-Carlo type dynamics [7, 8, 17, 40]. A system considered by Saad for instance comprises *N* Ising-like spins interacting on sparse and densely connected networks, driven by two interacting processes *σ* and *τ* with linked update probabilities (p.032131–2 [59]) *Pσ*_*i*_(*t* + 1) and *Pτ*_*i*_(*t* + 1) that have exponential functions of a common “temperature” and various spin interaction terms where all the sites re updated independently. The results show (Fig 1 [59]) the apparent co-existence of both equilibrium and NE domains; this was a preliminary study because no thermodynamical criteria were provided, nor theorems adduced to describe rigorously the nature of the coexistence; in standard thermodynamical equilibrium theory for instance, the phases can be defined, and there is the equality of the chemical potential for species in phases that are in equilibrium. One pertinent observation here is that for our purposes, one must be able to chose the simplest possible system that will not express complex phenomena/phase transitions that will make the variables or factors involved in the analysis inadequate, such as the discussion about the Maxwell demon and Feynman device demonstrates. The “updating” procedure in the recoverable trajectory, on the other hand conserves the probability density **P** during the coordinate transition **q**(*t*) → **q**(*t* + *δt*), where *δ* **P** = 0 for each streamline of channel *i*. Thus, if the NE entropy $S_{NE}$ can be written $S_{NE}=S_{NE}\left(P\right)$, then the transition is recoverable since $\delta S_{NE}=0$ along the updated trajectories; furthermore, the temperature is not constant unlike the Saad [59] system. Another work where there are temperature gradients is provided in [60]. As stated above, one objective of the work was to subsume the phenomenon of heat conduction within the Second law which BQ felt had not been articulated (p.21 [5]), where by definition, Fourier heat conduction conforms to the definition of heat energy transfer without any compensation, leading to zero efficiency conversion relative to the temperature reservoirs between the two points or regions between which there is energy transport by virtue of a temperature difference. In order to complete the identification of Fourier conduction with pure non-work heat transfer, in agreement with the Carathéodory definition, BQ also introduced the condition (p.13 [5]) **q** = 0 ⇒ ∇*T* = 0. Clearly this condition, in order to be compatible with Carathéodory’s definition of heat demands the fact that isothermal heat transfer is realized as a result of minute temperature gradients that can be introduced in either direction, and does not constitute another energy type or category according to this Carathéodory definition. In particular, all systems and mechanisms that admit of conversion had to be eliminated as referring to heat transfer, including any that locally seemed to violate the C statement of the Second law, such as the system report in [52]. If the C statement is violated locally between two adjacent mass segments of differing mass, then it becomes mandatory to interpret the movement of heat against a temperature gradient as due to a conversion process as in the case of Peltier conversion in thermoelectrics. The study of Alamino et al. is very interesting from the above point of view because for exactly the same reason the energy transferred cannot be classified as pure conduction if the C statement is violated locally between two surfaces. A plethora of different interaction mechanisms have been described in [60], where it is admitted that the critical definition of temperature “has remained an open question for a long time” (First par. [60]). We adopt the BQ definition of temperature by recourse to their diathermal fiber that obtains for both equilibrium and NE systems, but which seems to be not defined in these mathematical model studies; [60] are interested in the magnetization regime under mutual heat flux exchange of two adiabatically isolated surfaces, which cannot in any way resemble the two systems considered here in the Section below, since our lattice systems are not adiabatically isolated. Similar to previous work [59], in this study the two planes *σ*, *τ* respectively are updated for each spin state on the plane (Eqs (1–2) [60]) according to the Monte Carlo-like probability (for the *σ* plane) $P\left[\sigma_{i}\left(t+1\right)\right]\propto \text{exp}[\beta_{\sigma }\sigma_{i}\left(t+1\right)h_{i}^{\sigma }\left[\sigma \left(t\right),\tau \left(t\right)\right]$ with an interactive “Hamiltonian” with form $h_{i}^{\sigma }\;\frac{J_{\sigma }}{N_{\sigma }}\sum_{j\neq i}\sigma_{j}+J_{\sigma \tau }^{\prime }\tau_{i}$ with similar expressions for the *τ* plane. Also, there is a set of spin particles on the plane, which is to be contrasted with our single particle on each lattice site in our model; even unequal masses [52] leads to a contradiction of the C form of the Second law, unless one can identify it as a subsystem of a system where the C Second law statement is obeyed; thus complexity has to be interpreted thermodynamically in such a way that the laws of thermodynamics are maintained. The actual rate of heat flow and its fluctuations are not the major focus in their studies [60]. Of interest is the period two cycle in spin magnetization *M*_*σ*_ oscillating from +1 to -1 in time (2 in reduced units). A phase diagram for various interaction strengths across the two planes is mapped out (Fig 4 [60]). These studies show how the introduction of complexity can obscure the C Second law statement if there is a energy mapping between the magnetization states and net energy flow interpreted as “heat” flow “across two planes of Heisenberg spin lattices” for fixed and differing “temperature” parameter *β* against a temperature gradient unless one can also locate some compensating mechanism in the “environment”, which must also be characterized. Therefore, in order to describe conductive heat flow in terms of a disintegrating engine in this work, the system must be reduced to the simplest form of interactions whereby the Clausius heat transfer criterion holds between two thermal reservoirs; the literature references here do not conform to such a criterion unless compensating mechanisms are also brought into the argument that would preserve the truth of the Clausius statement. Similarly, for a more microscopic treatment of diffusive flux current [61] the temperature parameter so essential for characterizing heat flow is not prominently featured in the derivation of the additivity principle (Eq (10) [61]) which refers to coupled subsystems being independent but where at the point of contact the density maximizes a certain probability current (par. after Eq (10) [61]) *P*_*N*+*N*′_(*q*, *ρ*_*a*_, *ρ*_*b*_, *t*) where the *ρ*’s refer to the densities of the subsystems and *q* a type of time current density where the integrated current *Q*_*t*_ = *qt*. Indeed, the tacit assumption seems to be a system with a fixed temperature parameter if the *G* density (Eq (17) [61]) is Gaussian and is related to the canonical ensemble. By utilizing the BQ fiber concept, the center of mass motion of an ensemble of *n* particle can be characterized by a temperature *T* where $\frac{3}{2}kTn=<k.e.>$ if the diathermal fiber is connected to a reservoir at thermal equilibrium of known temperature *T*; relative to some other standard one would have *T* = *g*(<*k*.*e*.>, *n*). In any case, the total thermal energy current density *Q* would have the form <*Q* > = < *k*.*e*.> + <*V*>. It is not apparent how the fluctuations in diffusive flux in general can be related to Fourier heat conductive flux theoretically, since one would have to map particle number flux to a single localized particle having a spectrum of energy values for localized systems, and also relate a functional form of particle density with an entire spectrum of energies with a particular localized particle and its temperature. Indeed, it could be that the failure of the “caloric theory of heat” [84] is in part related to investigators being able to maintain and conceive of heat as some type of diffusive “material substance”. Thus detailed theories of diffusive current fluctuations in NE systems may not be applicable to heat transfer processes unless particle distribution ↔ energy density mapping functions are uniquely described; so far, this area seems not too well explored, if at all. Despite the lack of detailed mapping theorems, some scaling theory or relationship has been derived to relate diffusion to heat conduction recently [85], where it is claimed that if the mean square deviation in 1D diffusion is given as 〈*Δx*^2^〉 = 2*Dt*^*α*^, (0 < *α* ≤ 2), then the thermal conductivity scales as *κ* = *cL*^*β*^ where *L* is the size of the system, *β* = 2 − 2/*α* and if for normal diffusion *α* = 1, then *β* = 1 and Fourier’s law holds. In what follows, fluctuations are critical to our description, not as objects in themselves, (e.g. cumulant expansions (Eq (3–5) [61]) but in terms of net energy transfer within a stochastic loop Eqs (36 and 37). A relatively wholistic and comprehensive study of heat conduction phenomena relative to the current specialist literature is given for example in [63]; the work does not however discuss heat conduction from the point of view of the relation to the Second law in the manner found in the Carathéodory definition of heat flow as a response to thermal gradients and the topological structure of the phenomenon in phase space which he described. There is however, reference to ergodicity and phase space dynamics. The absence of proof of ergodicity for practically all heat conduction systems studied (2nd par., p.3 [63])—thereby having to *assume* convergence to average values—lead the authors to state at the very outset (Eqs (1–2) [63]) this tacit assumption in their discussion where they write “There are at least two distinct situations in which Fourier’s Law is observed to hold with high precision…” where the first situation is the conservation of flux or energy at any instant of time

$$\begin{array}{c} c_{v}\left(T\right)\frac{\partial }{\partial t}T\left(r,t\right)=-\nabla \cdot J=\nabla \cdot \left[\kappa \nabla T\right] \end{array}$$

subjected to specified initial conditions at time *t* = 0 of the temperature distribution and the second situation where the temperature of the heat reservoirs coupled to the system is invariant, where a stationary steady state is assumed (rather than proven) to exist with *no net matter flow* so that the heat current vector $\tilde{J}=\tilde{J}\left(r\right)$ is a function of position only, where $\nabla \cdot \tilde{J}\left(r\right)=\nabla \cdot \left(\kappa \nabla \tilde{T}\left(r\right)\right)=0$ and where $\kappa \left(\tilde{T}\right)\nabla \tilde{T}=Const.$ in one dimension. By showing that the continuous form of the Liouville equation does not exist in the standard form, where for a conservative Hamiltonian with partitioned coordinates

$$\begin{array}{c} H\left(p,q\right)=\sum_{i}\frac{p_{i}^{2}}{2m_{i}}+V\left(q\right) \end{array}$$ (26)

such as those that represent heat conduction systems (p.2, Eq (3) [63]) only a trivial solution obtains **q** = **C** *t* + *β* (Theorem 1, Corollary 1 [66, 67]), it was proved as a consequence that the celebrated recurrence theorem of Poincaré and Zermelo (p. 1102 [66, 67]) on which ergodicity and convergent time-averaging is based does not obtain in general. Similarly, the ergodic theorem of G.D. Birkhoff (p. 1109 [66, 67])

$$\begin{array}{c} \left\langle A\right\rangle =\lim_{C\to \infty }\int_{0}^{C}f\left(P,t\right)dt \end{array}$$ (27)

that provides a limit a.e. 〈*A*〉 of a function on a domain *V*, for all points *P* ∈ *V* for *V* defined by the Hamiltonian coordinates is dependent on the truth of the continuous form of the Liouville equation (p.20, Lemma 1 [86]) and therefore must also be subjected to a re-examination. Interestingly, Bonetto et al, state that local thermodynamical equilibrium is based on strong ergodic properties (2nd par., p.3 [63]) and that such properties have only so far been proven only for systems evolving via stochastic dynamics, and even here the relevant conserved quantity is usually the particle density rather than the energy density [87]. The only Hamiltonian system for which a macroscopic transport law has been derived is for a gas of non-interacting particles (p.3 [63]), which is not the system described here nor in all the major references provided here. Work on thermal conduction nevertheless goes on despite any proof of ergodicity being forthcoming. The main concern in this work is not focused on ergodicity, and the results are not dependent on the condition holding with regard to the zero entropy trajectory, which follows from the conservation of probability, and not from the Hamiltonian phase space pathway; further, the shape or form of the probability density profile need not be necessarily Gaussian; the basic requirement is that the entropy S has a functional form S=S[P] for a probability density *P* where for each energy type i, *δP*_*i*_ = 0 which is the condition that is fulfilled here, since each event of a collision is an energy interaction characterized by *i*. Heuristically, if the entropy were dependent on the kinetic variables *κ* independently of the probability, i.e. S=S(κ,P) then the Boltzmann definition of entropy as related to arrangement of microstates only would be compromised. Hence we deduce *P* = *P*(*κ*). We also note that the shape of the probability density or probability of states is arbitrary as long as there is no change along a state or event streamline (Eqs (24–27), p.168 [9]).

Also, for this particular representation, the temperature is that other quantity that needs to be characterized, and the kinetic energy and its relation to temperature is the key principle utilized in the entropy formulation. Dhar [53] states that there has been no rigorous derivation of the Fourier law expressed (Eq (1) [53]) as **J**(**x**, *t*) = −*κ*∇*T*(**x**, *t*) “starting from a microscopic Hamiltonian description”. The objective here is *not* to derive the Fourier law from the microscopic Hamiltonian but to show that heat conduction is compatible with the recoverable transition, and that the Fourier principle can be subsumed by the Second law, which is to be contrasted to the view of BQ that **F** stands external to it. It could well be that [88, 89] the Fourier law is only the first term in a power series expansion—where **F** stands independently of the order in the expansion (p.13, 3rd par. from bottom [5])—and that anomalies with that first order form is due to this incomplete description, including the anomaly that *κ* → ∞ whenever ∇*T* → 0 for fixed *N*, the lattice chain length, and for anomalous diffusive transport *κ* ∼ *N*^*α*^, *α* ≠ 0. where the value of *α* is system dependent; for instance for 1-D unequal mass distribution of gases (p.502, Fig 1 [53]) *α* = .32 for a mass ratio *A* = 2.62. Anticipating results in later sections, Dhar [53] has defined the local temperature *T*_*l*_, *T*(*x*) using the local or continuous approach for 1D (Eq (23) [53])

$$k_{B}T_{l}=\langle \frac{p_{l}^{2}}{m_{l}}\rangle$$ (28)

$$k_{B}T\left(x\right)=\frac{\langle \sum_{l}\left(p_{l}^{2}/m_{l}\right)\delta \left(x-x_{l}\right)\rangle }{\sum_{l}\delta \left(x-x_{l}\right))}$$ (29)

where the discrete form will be used, which is also equivalent to the continuous form if the time sequence index is *l* and integration is over the space-time coordinates. The definition of temperature is a strictly rigorous result for any Hamiltonian (p.475 [53]) from equipartition of the kinetic energy (k.e.) only, with no reference to the potential terms. Much of the review in [53] is devoted to calculating kinetic coefficients, such as the use of the Green-Kubo formula for thermal conductivity that requires fluctuational averages

$$\begin{array}{c} \kappa =\frac{1}{k_{B}T^{2}}\lim_{t\to \infty }\lim_{L\to \infty }\frac{1}{L}\int_{0}^{t}dt\left\langle J(0)J(t)\right\rangle \end{array}$$

of the heat current vector J(t). The Green-Kubo and allied methods require the use of time reversibility properties that were proven to be open to question [8]; but here we are not interested in determining the kinetic coefficients etc. that comes from complex algebraic procedures but in examining the nature of heat flow in relation to the Second law, and to clarify the BQ assertion that **F** is independent of the Second law. Here we show that the Second law actually subsumes the phenomena of Fourier heat conduction in a pathway that is mechanically irreversible, but thermodynamically reversible in terms of zero entropy generation about the defined path. These two concepts—mechanical and thermodynamical reversibility—are not viewed in contrary terms in practically the entire literature devoted to analyses. Further, it is implied here that the flow of heat occurs in such a manner that there is a conflation of the same process in both adiabatic and heat transfer processes, whereas in the conventional description of the Second Law using Carathéodory ideas, a loop integral traversing orthogonal adiabatic surfaces that do not overlap and isothermal surfaces (Sec. 8.8, p.201 [43]) is utilized to prove the existence of the entropy function of state. The intense work in finding solutions to differential, integral and algebraic equations centered about heat transport phenomena are not focused on the general thermodynamical structure of the phenomena as judged by the available literature. Further, the methods used, especially in the determination of the kinetic coefficients require assumptions, where (p.469 [53]) it is stated that “although these derivations are not rigorous, they are quite plausible…” but Dhar also cautions that the method assumes the PLE and also states that (p.470 [53]) “There are several situations where the Green-Kubo formula… is not applicable.” The use of the heat diffusion equation for instance (p.469 [53]) *S*(*x*, *t*) demands *S*(*x*, *t*) = *S*(*x*, −*t*), which requires the use of questionable time reversibility for the correlation function. This work does not correspond to the focus and topics pursued by most of the workers cited here. We do not make any major assumptions, apart from the integrity of certain expressions linking temperature to energy, such as derived from the RLL method (see below), which are considered to be exact expressions. Gedanken (G) experiments using the BQ “diathermal fiber” [13] leads to the same conclusion as demonstrated below. Using the (harmonic) Hamiltonian of the RRL method [90], written (p. 473, Eq (37) [53]) in the form

$$\begin{array}{c} H=\frac{1}{2}P^{T}{\hat{M}}^{-1}P+\frac{1}{2}X^{T}\hat{\Phi }X \end{array}$$

it was deduced that for white noise coupled reservoirs, (p.475 [53]), the local Kelvin temperature *T*_*l*_ for species *l* was given by

$$\begin{array}{c} \left\langle \frac{p_{l}^{2}}{m_{l}}\right\rangle =k_{B}T_{l} \end{array}$$ (30)

and because 〈(*d*/*dt*)*x*_*l*_ *p*_*l*_〉 = 0 this result for the definition of the temperature was valid for *any* Hamiltonian system according to Dhar. We henceforth refer to this result as the Dhar-RLL condition, which we here adopt as our method of determining the Kelvin temperature, which we also equate as an axiom to the temperature that would obtain if the BQ diathermal fiber were used experimentally to determine the temperature as described below. If we connected one end of a BQ diathermal fiber to a region of molecular dimensions of a system (*sys*) to probe its temperature in a G experiment, and the other end to a thermal reservoir at equilibrium (*res*) with temperature *T*_*res*_, where the potential of the system *V*(**r**_*sys*_) at the point of contact of the fiber to the system is set equal to the reservoir, *V*(**r**_*sys*_) = *V*(**r**_*rev*_). The fiber is designed to be made up of non-compressible thin rods that slide on each other without friction from the surfaces of *sys* to *rev*, with conservative site potentials inside the fiber to maintain its integrity. Also we may allow the fiber to transfer all the kinetic energy of a particle in either *sys* or *res* that interacts at the surface of the fiber to the other system that it is connected to, and the mean rate of collision or interaction of the reservoir with the BQ diathermal fiber is adjusted so that it equals that of the system per unit area. Then by hypothesis and definition, the temperatures of *sys* and *res* are the same if the net transfer of energy *sys* → *res* = *Q* and *sys* → *res* are the same for a time greater than the relaxation time *τ* of the interacting system; technically it may be more correct to state the condition as applying if ∑_*i*_ *δQ*/(∑_*i*_|*δQ*|) → 0 as the time *t* → ∞ for the heat increments transferred *δQ*_*i*_ over the time sequence in this G experiment. As pointed out in (p.468 [53]), the convective parts of the motion must be subtracted out in many of these current terms. A solution whereby a net zero energy transfer will occur is when the probability density *P*(*E*) with respect to the kinetic energy *E* satisfies *P*_*sys*_(*E*) = *P*_*res*_(*E*) and *P*_*res*_(*E*) is a Canonical distribution. However, even if this is experimentally not the case, then over a time duration larger than the relaxation time, the total average kinetic energy transferred in either direction must be the same, and therefore the average k.e. per molecule which is reflected in the balance equation must have the relations *Q*_*sys*_ = ∫_*Ω*_*E*__ *EP*_*sys*_(*E*)*dE* = ∫_*Ω*_*E*__ *EP*_*res*_(*E*)*dE* = *Q*_*res*_ for the quantity of kinetic energy “heat” per particle. This is the same equipartition result as for the RRL method as extended by Dhar to any Hamiltonian (Sec. 3.1, par. before Eq (49) [53]). In our reference model, we note that the change of energy during a collision for the same potential energy for both particles involved Eq (48), and so the “equipartition” results for the kinetic energy of an equilibrium system are valid within this representation for the NE steady state system as well. Indeed, it is explicitly mentioned that the potential energy equipartition result is of no relevance to the model of heat conduction and that it is included to suggest that for even more complex phenomena, the potential terms might contribute to the overall dynamical structure. The Dhar observation of having to subtract the convective kinetic energy is maintained here by placing the fiber at the equilibrium plane when the potential energy is zero as shown in (Eqs (51,52)). Indeed it is shown by the G experiment that the potential can be incorporated into the kinetic equipartition result exactly (Eqs (51,52)) for a particular system with an arbitrary conservative potential $V_{c}$

In terms of stochastic analysis, there are descriptions of temperature invariant systems which attempt to correlate the mesoscopic and macroscopic aspects of non-disintegrating systems [64] where full description of the thermodynamics may be attained by addition of reversible heat from the stochastic quantities such as $d^{\prime }\tilde{Q}$ to derive a measurable heat $d^{\prime }{\tilde{Q}}_{m}$ by a transformation or mapping process, e.g. $d^{\prime }\tilde{Q}\mapsto d^{\prime }{\tilde{Q}}_{m}\equiv d^{\prime }\tilde{Q}-Td\frac{\partial \tilde{F}}{\partial T}$. There is no resemblance of these stochastic trajectory analysis and its relation to the macroscopic body to the concepts being developed here to encompass or subsume heat flow within the Second law as defined by BQ; the focus is not on the same topic.

An example of a locally homogeneous system that would comply with the extended Clausius statement of the Second law $C_{e}$ is the structured assembly of a 1D lattice chain of identical masses with the same conservative intermolecular potential between adjacent sites *i* and *i* + 1, where the particles have a mean time independent position whilst involved in the process of transferring energy between the two ends of the lattice chain that are maintained at different temperatures. A thermodynamical model is developed here that incorporates the above recoverable trajectory for such a non-disintegrating lattice chain. Since attempting to model such a process is a very involved task, with possibly unproven assumptions being surreptitiously imported into the creation of the theoretical structure that could be simulated and computed, the following methodology is utilized to ensure as far as possible that no extraneous concepts are imported:

1. Identify a discrete non-disintegrating model system that, from theoretical considerations would have to conform to the above process of a recoverable process whilst conforming to the extended Clausius Second law statement $C_{e}$
2. Apply that model to the continuous lattice chain, even in a restricted subsystem of a single particle in the lattice chain to extend concepts that would have to be used for a more comprehensive treatment of irreversible phenomena
3. Quantitatively check the model for numerical agreement with total heat flow terms
4. Extend the model from just one particle interaction with the adjacent pair to longer ranges of interactions so that one might be able to predict the kinetics and thermodynamical variable profile over the entire lattice
5. Lastly, from the above, it may be possible to construct a more comprehensive and extensive thermodynamical theory by generalization of the above item (4) that might be able to encompass both equilibrium and NE interactions.

Here we describe results for items (1–3) above where the experimental result is consistent with the theoretical paradigm: items (1–3) illustrates feasibility and proof of concept. The next step would be to extend the 1-particle interaction description developed here to multiple interactions. The other items in the methodology list (4 and 5) are project proposals that has the scope of creating a paradigm that subsumes various classes of processes and transitions mentioned previously (adiabatic, isothermal, reversible and irreversible).

It was demonstrated before [7, 8, 17] that the pivotal concept of time reversibility is mathematically incorrect in its major applications to wide-ranging phenomena, and that these fallacies have been incorporated into mainstream thermodynamical interpretation of the various classes of processes and transitions (e.g. see pp.36, 52, 141–7, 356–7 [91]).

**Fig 2:**
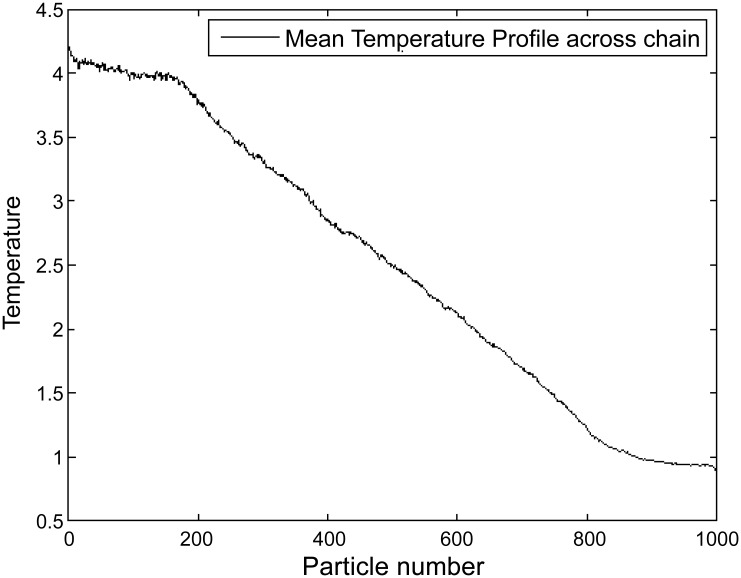
Temperature Profile across chain. From Table 1, the *u.s.d*. for the freely vibrating particles is of the order of 0.14 whereas the thermalized particles 1–200 are maintained at the average temperature T = 0.40014E+01± 0.8E-02, and the colder themalized particles 800–1000 have the average temperature T = 0.99987E+00± 0.2E-02.

The basic ideas associated with the Carnot cycle with its ‘reversible’ pathways has not escaped characterization based on these so-called ‘reversibility’ postulates, and hence the above remarks require some qualification. The original work of Carnot [92] describes essentially a work cycle whose work energy variable *w* is not a perfect differential and where the introduction of heat (Figs 2 and 3, p.70 [92]) into the working substance involves the transfer of heat about vanishing temperature gradients where there is *“… no contact between bodies of sensibly different temperatures”* (p.68 [92]). More recently, work have attempted to incorporate adiabaticity in thermal heat transfer [93] coincidentally along similar lines much earlier (e.g. Sec. 4.3.1 “Verification of the theorem for an adiabatic process for a perfect gas”; Sec.6. where it is stated that “It appears that the problems that remain in this paper are the working out of the details of the kinetics of energy transfer, for instance *in relation to the Fourier heat conduction law*….” [9]) but in (e.g. Eq 12–13 [93]) there are irreversibilities that arise from discontinuous velocities of their system of interlocking pistons with discontinuous temperature variations of the systems between the pistons, and where there is work transfer contact between bodies of sensibly infinitesimally different temperatures, which contradicts the Carnot assumptions, and which is avoided in the current development where purely dynamical systems with specified Hamiltonians are studied without adiabatic temperature discontinuities. Also, a net stationary frame of reference is involved where the internal source term *i*(*x*) ≥ 0 (Eq (58) [93]). The assumptions made in applications of Carnot’s analysis (p. 133, R. Clausius, “On the application of the theorem of the equivalence of transformations to the internal work of a mass of matter” [18]) encouraged views developed in the previous centuries to conceive heat as a degraded form of energy that increased the entropy of a system by traversing a thermal gradient from hot to cold (thus increasing the entropy of the system), as seen for instance in (Eq (58) [93]).

Indeed the calorimetric definition of heat (1968 5th Ed, p.73 [19]) is *“…that which is transferred between a system and its surroundings by virtue of a temperature difference only”*.

Carathéodory conceives of heat (J. Kestin ed., Introduction, p.229 [1]) in the following manner: “‘*Furthermore, when two bodies of different temperatures are brought into contact, heat always passes from the hotter to the colder, and never in the reverse direction*’”. Here we shall show for recoverable transitions, which includes heat conduction, the energy transferred is actually “work” in the local sense, although the movement is from hot to cold, in accordance with previous definitions. The degradation idea is also evident in É. Clapeyron’s 1834 work (p.38 [94]):*“…in any mechanism designed to produce motive power from heat, there is a loss of force whenever there is a direct communication of heat between two bodies at different temperatures and it follows that the maximum effect can be produced only by a mechanism in which contact is made only between bodies at equal temperatures”*. This coupled with the definition of heat above lead to the concept of “reversible transfer”, where the concept of “reversibility” is a rationalization imported from mechanics with its belief in the reversibility of its laws in classical and later in quantum physics and thermodynamics [91]. The derivation of the Onsager reciprocity relations and the Boltzmann H-theorem are examples of misapplications of such time-reversible and Liouville equation assumptions [7, 8, 17, 66, 67].

Finally, it was proven that the pivotal Liouville equation, derived from the Hamiltonian in (**p**,**q**) Liouville phase space is in general not valid as a continuous equation, where a stochastic analog of the same form was derived in its place [66, 67]. In particular, attempts to deduce zero-entropy paths from Liouville space were shown to be flawed (see references therein at [66, 67]). It was expressly stated in [9] that the zero-entropy “recoverable trajectory” developed there is *not* described in Liouville space (p.169, top par. [9]), and that further, as illustrated in the current work, there is a stochastic back-scattering of energy involving a non-conservation of energy about a stochastic work cycle that at first sight would not readily follow from a standard mechanical Hamiltonian using continuous, non-stochastic variables. If such Hamiltonians H(p,q) are used in describing NE mechanical systems, then assuming a general average $\left\{\bar{p},\bar{q}\right\}$ for all coordinates *j* where *j* ≠ *i*, then $\oint \frac{\partial H}{\partial q_{i}}\left(q_{i},\bar{p},\bar{q}\right)dq_{i}=0$ which is not observed in the simulation results obtained here, implying that the introduction of hybrid elements (random energy impulses at the ends of a chain of vibrating atoms in this case) to simulate thermostated regions for instance destroys the continuum description of the mechanical Hamiltonian and that, in addition some very complex cooperative phenomena may be involved with the standard Hamiltonian where the assumption of averaged coordinates do not apply. In particular, thermodynamical systems involving averaged variables—as in thermodynamics—cannot be described by such primitive mechanical Hamiltonians; and if used, theorems would have to be created to relate the mechanical phase space of the Hamiltonian to the averaged motions of particles characterized by the respective phase space [95].

The conventional description of conductive heat transfer describes the transport of “heat” energy through assumed reversible dynamical laws, and which involves a positive local internal entropy production rate *σ*, so that ∫_*V*_ *σdV* = *dS*_*i*_/*dt* ≥ 0 [96]. If the entropy vector has a component **J**_**s**_ = **J**_**q**_/*T* (**J**_**q**_ being the heat current vector), then at the steady state

$$\begin{array}{c} \nabla .J_{s}=-J_{q}.\nabla T/T^{2}\neq 0 \end{array}$$ (31)

in a temperature gradient with a non-zero heat current present and indeed in conventional descriptions, *σ* has the above component due to the conductive heat contribution (Ch.III, p.24, Eq (21) [96]). One important aspect of the expressions for *σ* is that it allows for the identification of the Onsager reciprocity coefficients that couple forces and fluxes (Ch.IV, pp.33–36 [96]). Previously, it was remarked (p.163, Sec.2 [9]), in keeping with the Clausius definition, that for closed systems a more appropriate definition would be $\dot{S}=-\int_{V}\left(\nabla .J_{q}/T\right)dV$ which would yield zero entropy production apart from the system boundaries. Further, BQ have argued that Fourier’s inequality (following Eq (31) with **J**_**q**_ = −*κ*∇*T*), embodying **F**

$$\begin{array}{c} \kappa {\left(\nabla T\right)}^{2}/T^{2}\geq 0 \end{array}$$ (32)

is a local principle, not subject to the Second law since the latter refers to global work-heat transitions, and may well be an independent principle.

Recall that the modern Carnot cycle is strictly developed with 2 types of heat transitions of the working substance, the “isothermal” and the “adiabatic”.

One conclusion of Carathéodory thermodynamics [20] for “‘macroscopic’” systems is found in Axiom II: *In every arbitrary close neighborhood of a given initial state, there exists states that cannot be approached arbitrarily closely by adiabatic processes*.

In what follows, we show that relative to a particular subsystem, within the heat conducting chain constituting atom *i* and the adjacent one *i* + 1, pure heat energy transfer (according to the Carathéodory definition of heat) occurs and in accord with conventional definition, but at the same time according to the major assumptions of recoverable transitions [9] where the following conditions obtains:

1. A net adiabatic process in the forward direction occurs
2. The energy transfer being the work done, conventionally considered the heat increment
3. The work is mechanically irreversible, but thermodynamically reversible (zero entropy) where the micro-transitions complete a Carnot-type loop within the subsystem
4. The stochastic integral for the energy about a loop is not zero, implying that the mechanical Hamiltonian is non-conservative in such hybrid systems
5. There is a distant implication that both adiabatic and isothermal processes may be unified, at least at the micro-level, which would require answering challenging questions as to how macroscopic descriptions, such as due to Carathéodory, emerge from the micro-processes, which encompasses both adiabatic and isothermal ones.

The above seems to indicate that if the direction of this research project is deemed reasonable, then there are gaps that need to be bridged between some of the more conventional definitions and the deductions that are being made here as detailed and itemized in (1–5) above, since for example a vectorized work term appears as ‘heat’ in this representation, the defined entropy change is zero (thermodynamically ‘reversible’) with mechanical irreversibility along the dynamical trajectory, with non-conserving Hamiltonians for averaged coordinates. Another added complexity to this and other thermodynamical description is the assumption of ‘local’ equilibrium raised to the level of a ‘Principle’ [21, 97, 98] for fundamental applications. This principle was proven mathematically and via simulation to be incorrect [22, 23]; in particular the eminent works of B. C. Eu, García-Colín and their followers e.g. [99] where some “uncompensated function” *N* was introduced to create a perfect entropy differential $\frac{dS(t)}{dt}$ for irreversible processes having the form (Eq 15 [99])

$$\begin{array}{c} \frac{dS(t)}{dt}=\frac{1}{T(t)}\frac{dQ(t)}{dt}+\frac{dN(t)}{dt} \end{array}$$ (33)

was explicitly disproved in (Sec.2, p. 838, **Proof** [100]) and shown not to be feasible [22, 23] on other physical grounds. This scheme therefore cannot be applied to heat conduction, even if there is some stationary conservation of entropy, since it cannot be applied to disintegrating systems, even if the theory were entirely correct. What is evident, however, is that this assumption of the PLE obtains for a large class of systems over extremely large magnitudes of forces and fluxes, which explains its utility, despite it being not an exact principle, but an (extremely good) approximation to the NE state functions using the equilibrium state functions without augmented variables. It is critical to note that the current work does not in any way conform to the Clausius Inequality and the sub-Carnot efficiency that it implies for real systems since the development here is based on a certain defined class of spatially non-stationary disintegrating systems that retains its optimum heat to work efficiency (p.177 [9]) at all stages of its trajectory.

One major objective of this work includes answering the significant question posed by BQ in their seminal study of heat conduction, the Second law and magnetothermoelectric phenomena [5] which has profound implications as to how thermodynamical systems are to be considered in framing new theorems, as they have done. Their views include two of thermodynamical significance (p. 9, bottom par. and p.21, top par. [5]):

- The flow of heat without compensation—argued as synonymous with the energy current flow associated with Fourier conduction—is a local phenomena (within the limits of material continuity) with no reference to the global statements of the Second law.
- The Fourier principle stands as a principle that is independent of the Second law.

This work qualifies the above axiomatics. Here, a paradigm is presented where within the limits of material continuity, the Fourier principle is local in nature but it also conforms to the Second law locally. We therefore show that it lies within the scope of the Second law. The formidable literature concerning heat conduction very briefly surveyed above is by and large not concerned with any of the above issues, especially on the most basic relations to the foundations of thermodynamics, which is at the core of the current work: there is an emphasis on Natural Philosophy, the area within which Kelvin preferred to define his prodigious labors in physics, where he refused the designation “physicist” [5], preferring the term natural philosopher. This was because he was interested more in ontological questions, such as the framing of hypotheses and laws, which were predominantly not, even in his time, the primary occupation of his peers, who were preoccupied with solving various mathematical problems based on the axioms and laws structured by natural philosophers such as Kelvin.

Some obvious assumptions used in this work is put forward as axioms. They include the following:

**Axiom 1** *Temperature parameters may be ascribed at the steady state to each of the oscillating particles along the lattice chain based on the extended Zeroth law*

**Axiom 2** *The equilibrium classical density-in-phase distribution and the probability distributions for Fermionic and Bosonic systems are unique for any particular temperature*.

**Axiom 3** *For systems probed by the diathermal fiber, the thermostatic temperature of the fiber is the same as the steady state system temperature, and corresponds to the density-in-phase distribution for that temperature for the system concerned with a specified Hamiltonian*

Axiom (1) refers to the fact that the temperature parameter is stilled used as a significant thermodynamic variable for nonisothermal NE systems. Here, we focus on the Fourier law that assumes that the temperature variable and its gradients can be used to characterize heat flow, as also is the presupposition in the definition of heat in thermostatics and in Carathéodory thermodynamics. Since our characterization of temperature uses the BQ diathermal fiber connected to a thermal reservoir in thermal equilibrium, Axiom (2) ensures that our temperature parameter is unique for a NE system. Axiom (3) justifies so much theory [53] that couples system Hamiltonians with the temperature parameter. We use the Dhar-RLL deduction to relate the theoretical temperature to the BQ method of determining NE temperature, using the Axiom that both the Dhar-RLL temperature is equal to the BQ experimental criterion. We do not utilize the concept of the PLE in the traditional sense here, since two temperatures are associated with a particular particle or scattering site, and where the BQ concept of temperature that couples an equilibrium reference system of known temperature to a NE system via a “diathermal fiber” where whilst a temperature might be specified, the energy distribution need not be Boltzmann for both canonical and non-canonical coordinates. The only use of “equipartition” here is the Dhar-RLL result which seems to also follow from the G experiment above, which is applied to the deconvoluted processes of forward and backward scattering interactions, which means that the time segments admit discontinuities. These phases of the trajectory may be sampled in principle by the diathermal fiber, and by the Axioms above (1–3) each of the processes would be characterized by the temperature *T*; for a single particle over *N*_*b*_ time averaged readings, or for a single instance of time for *N*_*b*_ particles $T=\langle \sum_{i=1}^{N_{b}}{p_{i}}^{2}/\left(2m_{i}\right)\rangle =\hat{T}$ for masses *m* and momentum *p*.

## Materials and Methods

Below the computational methodology and the equations and theory on which it is based is developed.

### Description of the simulation system

The results presented here refer to a 1000 linear atomic chain, labeled 1 to 1000 (where it might be envisaged as a horizontal structure) from left to right, with the first 200 atoms on the left thermostated to 4.0 (reduced units) whilst atoms 800–1000 were maintained at 1.0. The method of thermostating used a classical, non-synthetic algorithm developed or popularized by Hafskjold and Ikeshoji [101] where the thermostated atoms were scaled according to ${\dot{q}}_{i}^{\prime }=\alpha +\beta {\dot{q}}_{i}$, with *α* and *β* common to all relevant atoms to maintain the temperature *T* where $T=\frac{1}{\left(1+N_{b}-N_{a}\right)}\sum_{i=N_{a}}^{N_{b}}m{\dot{q}}^{2}$ and in reduced units, *m* = 1; *N*_*a*_ is the initial index of the particle and *N*_*b*_ the last for the respective thermal reservoir. Here and elsewhere, the validity of the Dhar-RLL theorem for the determination of temperature for the deconvolved processes is assumed. One operational definition of temperature requires the use of a ‘diathermal fiber’, according to the method of BQ [13] for NE systems. These assumptions give the form of the probability density for the equilibrium portion of the fiber as *ρ*_*i*_, as $\rho_{i}=\Theta \text{exp}\left(\frac{-H(p_{i},V)}{kT}\right)$ (p.41, Eq 7 and p. 63, Eq 9 [102]) where the *T* is the same as for the region of the NE system coupled to it. We have shown from the elementary FP equation that there is either distortion or rescaling of the temperature for the entire process; on the other hand, for separated processes in NE systems with the BQ fiber used to determine temperature, the use of this density refers to the mean total energy interchange with the diathermal fibre connected to a system in equilibrium; the exact density for the combined potential and kinetic energies need not be MB as demonstrated in the G experiment. This mean energy is related to the “thermal quantum” that was mentioned previously (Theorem, p.239 [10]) in another context. No coherent theory exists that is able to unify the concept of temperature for both the equilibrium and NE regimes exactly, nor is a mathematical definition of NE temperature forthcoming; hence we use the operational BQ method to determine temperature. One the other hand, the use of these densities for NE regimes in Linear Irreversible Thermodynamics and in the calculations of transition probabilities (p.553, Eq (15.2.10 [33]) must be contrasted to recent developments of NE densities [34] and the basic FP development for transients already alluded to. Currently, this work assumes the mode of transfer to be due to the kinetic energy changes during collisions and Dhar deduces that such a density is applicable to all Hamiltonian systems with the condition (p.475 [53]) $\langle \frac{d}{dt}x_{l}p_{l}\rangle =0$ for the definition of temperature as described above; we showed from G experimental considerations that this is the case irrespective of the density distribution. Also we have partitioned the processes and the temperatures obtained pertain only to the various partitioned processes can be enumerated in a discontinuous time sequence and which are probed by the BQ fiber since one must define the various processes as belonging to a particular scattering channel, and sampling is done relative to the data in each of the channels which is absent in the traditional analysis. The thermal energy content is widely defined in continuous and conservative systems as $\bar{H}\left(p_{i},q_{i},V\right)$, H the Hamiltonian and *V* the coordinate dependent potential. For our discontinuous system partition, both the average kinetic energy and potential limits exists by measurement, and the mean thermal energy of the system <*Q*> is defined to be (for equal mass systems)

$$\begin{array}{c} <Q>=\bar{H}\left(p,q\right)=p.p/2m+V\left(q\right) \end{array}$$ (34)

for each of the scattering channels.

As is well know, the harmonic potential only [90] does not yield the expected Fourier heat conduction law **J**_**q**_ = −*κ*∇*T*, with near constant *κ*, but one might also say that for the harmonic interaction potential between particles, the thermal conductivity *κ* is a very sensitive function of the temperature, and that it is also not a local property but may be a function of the entire temperature distribution. Hence there is no reason to suppose that the Fourier heat conduction law breaks down for Harmonic potentials. These and others are very interesting research questions, as Lebowitz et al. have testified to [63]. Here, the interparticle potential V between particles *i* and *i* + 1 in a lattice chain has been defined as having the following form by several workers:

$$\begin{array}{c} V=k_{h}{\left(q_{i+1}-q_{i}\right)}^{2}/2+b_{h}{\left(q_{i+1}-q_{i}\right)}^{4}/4. \end{array}$$ (35)

Here *k*_*h*_ = 1.0, *b*_*h*_ = 0.5 where these parameter values are chosen for good reproducibility, e.g. as determined by Tejal et al. (Fig 6 [55]). The *q*’s are the displacement from the equilibrium position with the separation distance of unity, and the force on particle *i* due to particle *i* + 1 is defined as $F_{i,i+1}=-\frac{\partial V}{\partial q_{i}}$. For such systems as a lattice chain, we define the *partitioned work* done *on* *i* due to the force from *i* + 1 as

$$\begin{array}{c} \Delta w_{i+1\to i}=\int_{t_{1}}^{t_{2}}F_{i,i+1}\;{\dot{q}}_{i}\;dt\approx m\oint_{stoch}F_{i,i+1}\;dq_{i} \end{array}$$ (36)

between the time interval [*t*_1_, *t*_2_], and the work done *on* *i*+1 due to the force from *i* as

$$\begin{array}{c} \Delta w_{i\to i+1}=\int_{t_{1}}^{t_{2}}F_{i+1,i}\;{\dot{q}}_{i+1}\;dt\approx n\oint_{stoch}F_{i+1,i}\;dq_{i+1} \end{array}$$ (37)

for large enough time interval *t*_2_ − *t*_1_ for *m*, *n* being integers. The above equations pertain to systems that oscillate, as with particles along a lattice chain, and the loop or circular integral given are over cycles (*m* and *n* respectively) where ∮_*stoch*_ refers to the average work done over the consecutive *m* or *n* cycles respectively. The equations become exact as *t*_2_ − *t*_1_ → ∞, i.e.

$$\begin{array}{c} \oint_{stoch}=\frac{\int_{t_{1}}^{t_{2}}F_{i,i+1}\;{\dot{q}}_{i}\;dt}{m} \end{array}$$

as *t*_2_ − *t*_1_ → ∞ for *m* consecutive cycles. The above equations normalized over unit time are defined thus:

$$\begin{array}{c} \delta w_{2\to 1}=\Delta w_{i+1\to i}/\left(t_{2}-t_{1}\right) \end{array}$$ (38)

$$\begin{array}{c} \delta w_{1\to 2}=\Delta w_{i\to i+1}/\left(t_{2}-t_{1}\right). \end{array}$$ (39)

We make use of the standard Simpson second order 3-point formula (fourth order error) numerical integration [103, 104] for the over 3 million (M) consecutive time steps of stepsize 0.001, equal to the MD timestep. Since all the particles are oscillating, particles *i* and *i* + 1 too can be viewed as oscillating back and forth an average of approximately *m* times (to the nearest integer) about their mean position. If this partition force were conservative, then

$$\begin{array}{ccc} \lim_{m\to \infty }\Delta w_{i+1\to i}/m & = & 0 \end{array}$$ (40)

$$\begin{array}{ccc} \lim_{n\to \infty }\Delta w_{i\to i+1}/n & = & 0 \end{array}$$ (41)

and the above limits for large values of time intervals are not observed in all the simulations carried out (see Table 1) with varying lengths from 3 to 10 M time step intervals, where, when these integrations of different time lengths are normalized to per unit time, yielded the same numerical quantities at the steady state. Hence these stochastic path integrals (involving a hybrid system of random energy and momentum impulses at the reservoir coupled to a standard system Hamiltonian without a time dependent variable) have non-conservative “‘Hamiltonians’” even if the classical Hamiltionian is continuous with continuous variables having no explicit time dependence. We note that distinguished theorists routinely use the Liouville and Hamilton equations in modeling these heat conduction problems e.g. [63, 90].

**Table 1:** The particle index number # is provided by the header on the *l.h.s*., with all the other variables (such as the *δw*’s) fully described in the text. The temperature of the particle # and that of the adjacent one on its *r.h.s*. with index #+1 appears in columns 4 & 5. Column 6 are the results for <*T*_*a*_> given by Eq (82). The *u.s.d* of the results for the *δw*’s is approximately 0.12 × 10^−1^ and that of the temperatures 0.14 × 10^0^.

part.#	*δw*_2→1_	*δw*_1→2_	temp. #	temp. #+1	<*T*_*a*_>
250	-0.21070E+00	0.21110E+00	0.35166E+01	0.35038E+01	0.35162E+01
300	-0.21031E+00	0.21046E+00	0.33159E+01	0.33037E+01	0.33154E+01
350	-0.20958E+00	0.20976E+00	0.31287E+01	0.31141E+01	0.31283E+01
450	-0.20978E+00	0.20988E+00	0.27131E+01	0.26945E+01	0.27127E+01
500	-0.21036E+00	0.21034E+00	0.24834E+01	0.24830E+01	0.24830E+01
550	-0.21185E+00	0.21182E+00	0.23192E+01	0.23078E+01	0.23187E+01
650	-0.21076E+00	0.21086E+00	0.18816E+01	0.18692E+01	0.18812E+01
700	-0.21149E+00	0.21146E+00	0.16796E+01	0.16676E+01	0.16792E+01
750	-0.21236E+00	0.21231E+00	0.14668E+01	0.14619E+01	0.14664E+01

### Formulation of Theoretical Reference Model

Assuming the validity of the Dhar-RLL definition of temperature to be valid for the deconvolved k.e. equipartition, this particular model will be shown to exhibit recoverable transitions in the manner described below. For what follows, two related models of heat conduction are discussed:

(i) a discrete case where there are no interparticle potentials, but only a quadratic site potential, and where energy transfer along the lattice chain is through elastic collisions between particles (described as the reference model)
(ii) a non-discrete system where the particles in the lattice interact via continuous potentials (Eq 35).

System (i) is the reference model, and because of its discrete nature of particle interactions, can be modeled as a recoverable process with less ambiguity compared to continuous systems. System (i) is then generalized to system (ii), where a 1 ↔ 1 correspondence is made between the variables in (i) and that of (ii), so that the factors involved in recoverable transitions for continuous systems may be identified. System (i) with its isolated potential is modeled as conforming to the extended equipartition concept of Dhar-RLL for the two scattering processes, with two associated temperatures about one particle label using the BQ diathermal fiber which is made to interact during one phase of the scattering process. It no net change of energy transfer is observed, and if each of the energy quantum transferred is monitored, then one can define both a temperature to that scattering process, and also test out the validity of the energy probability density function associated with the amount of energy transferred by computing the probabilities; the Zeroth law is invoked to determine its temperature. Although the reference model is here used only as a template, where its set average equilibrium separation distance *d*_*eq*_ is not a relevant variable when constructing from it the continuous model which is simulated, yet one can still, by equating the thermal k.e. energy at the equilibrium point to the maximum amplitude $kT=k_{h}d_{eq}^{2}$ to derive an estimate for the equilibrium separation distance,

$$\begin{array}{c} d_{eq}\leq {\left(\frac{kT_{m}}{k_{h}}\right)}^{1/2};\;\;T_{m}=\left(T_{h}+T_{l}\right)/2 \end{array}$$ (42)

where *T*_*m*_, *T*_*h*_, and *T*_*l*_ are respectively the mean temperature of the 1D uniform conducting material, held at the ends at temperatures *T*_*h*_ and *T*_*l*_.

Of note here is that the localized particle exhibits two temperatures as it interacts with adjacent particles; there is the mean kinetic energy temperature and the temperature associated with the transfer of energy from particle *i* to the adjacent particle (on the right, particle *i* + 1) in a basic adiabatic transition. Once the reference model is described, which involves discontinuous elastic collisions, it is then applied to the continuously interacting case that we validate by simulation; the key link to the continuum is to realize that each moment of time constitutes a “collision” interaction of the coupled particles relative to this “‘isolated’” reference model.

The theoretical reference model is “isolated” in the sense that its energy is well defined and localized except for the time of collision with elastic hard sphere energy interchange as shown in Fig 1(a) where they oscillate in the horizontal direction due to a site potential *V* acting vertically with a small horizontal projection which we can assume to be harmonic with regard to its displacement *q*_*i*_ from the horizontal equilibrium position at *q*_*i*_ = 0, (this assumption is not mandatory but it simplifies matters) and so the potential energy $V_{i}=\frac{k_{h}q_{i}^{2}}{2}$, the kinetic energy is $k.e.\left(i\right)=\frac{m{\dot{q}}_{i}^{2}}{2}$ and the total energy *E*_*i*_ = *V*_*i*_ + *k*.*e*.(*i*). The model is eventually modified to cover the situation in Fig 1(b) by appropriate choice of subsystem where even in the coupled state, one can conceptualize each particle as being isolated at each instance of time *t*. For what follows, particle *i* is referred to simply as *i*. The proof of the feasibility of this model is that a simulation of an allied system with the same dynamical structure—as will be demonstrated—has been described in detail [105].

A recoverable transition (Theorem and Eqs (85–86) [9]) considers the streamline

$$\begin{array}{c} \delta S=\delta \left(\frac{Q_{E}}{T}\right)=0 \end{array}$$ (43)

where *Q*_*E*_ is defined as for <*Q*> in Eq (34), i.e. *Q*_*E*_ = <*Q*>; the energy term *Q*_*E*_ is for the whole ensemble or for the time average following Gibbs for a single particle or system. The potential is *not* utilized to determine *T* the temperature, which is computed according to the Dhar-RLL rationalization where only the kinetic energy is considered.

Heuristic justification of why extended equipartition could be a ‘strong’ principle in multi-temperature, 1 particle representations that rely on the Dhar-RLL condition

The general classical Hamiltonian for any one particle

$$\begin{array}{c} H=\frac{p_{i}^{2}}{2m}+V\left(q_{i},\Omega \right) \end{array}$$ (44)

for process b (before) a collision and a (after) a collision would cover (*p*, *q*) phase space ∂*V*_*a*_ and ∂*V*_*b*_ of the overall space for all possible interactions *V*_*c*_, where *V*_*c*_ = ∂*V*_*a*_ ∪ ∂*V*_*b*_; **Ω** are the set of variables that pertain to the NE situation. If the set of eigenfunctions and energy eigenvalue for the quantum version of Eq (44) for processes a and b are {*ψ*_*a*,*i*_, *E*_*a*,*i*_} and {*ψ*_*b*,*i*_, *E*_*b*,*i*_} respectively, then for sufficiently large numbers of states, using the standard optimization techniques to derive the canonical distribution would yield for the steady state the probability distribution

$$\begin{array}{c} P_{a,i}=e^{\beta_{a}E_{a,i}}/Z_{a};Z_{a}=\sum_{i=1}^{M}e^{\beta_{a}E_{a,i}} \end{array}$$

with similar expressions for processes b. This argument presumes that an energy spectrum {*E*_*i*, *NE*_} exists for the Schrodinger equation ${\hat{H}}_{NE}\Psi_{NE,i}=E_{NE,i}\Psi_{NE,i}$ describing NE time-dependent steady states with nondegenerate energy eigenvalues and that the methods used for the equilibrium canonical ensemble also apply for the NE steady state simply because the equilibrium state is a special NE state with zero net fluxes. The canonical distribution is chosen in this particular instance for a single particle/system assuming that solutions exist for the Schrodinger equation and that the temperature *β* can be characterized via the diathermal fiber that is used as a probe to determine the temperature, and because the temperatures and eigenvalues are not the same, we have in general that *P*_*a*,*i*_ ≡ *P*_*b*,*i*_ ≡ *P*_*c*,*i*_. Averaging $\frac{p_{i}^{2}}{2m}$ by the canonical distribution yields the average kinetic energy $k.e._{a}\left(i\right)=\frac{kT_{a}}{2}$ and similarly for processes b, where *T*_*a*_ (*T*_*b*_) are determined by the diathermal fiber and is also the equilibrium temperature which is used to parameterize the system for processes a and b that can be distinguished. Similarly using time averaging and the Virial result the following equation obtains in general if the classical equipartition obtains for the momentum coordinates according to the Dhar-RLL condition Eq (30) for the left hand side of the following (p.80–83 [106]) equation $\bar{p_{k}\frac{\partial H}{\partial p_{k}}}=\bar{q_{k}\frac{\partial H}{\partial q_{k}}}$ meaning that the right hand side is equivalent to it. For the potential energy written $p.e.\left(i\right)=\frac{k_{f}}{2}q_{k}^{2}$ we derive from the kinetic energy and virial $<p.e.\left(i\right)>=\frac{kT_{a}}{2}$. We note that the quantum result for the steady state solutions to the Schrodinger equation {*E*_*i*_} has the NE variables, such as those pertaining to current flows and gradients, which are absent in the equilibrium system. Therefore, these results are not equivalent to thermostatic systems where there are no current flows nor gradients and the energy eigenvalues would include variables for the NE flow and gradient variables absent for the thermostatic situation. Because subsets for processes a and b are involved, it is evident that *Z*_*c*_ > *Z*_*a*_, *Z*_*c*_ > *Z*_*b*_, {*ψ*_*c*,*i*_, *E*_*c*,*i*_} ⊇ {*ψ*_*a*,*i*_, *E*_*a*,*i*_}, {*ψ*_*c*,*i*_, *E*_*c*,*i*_} ⊇ {*ψ*_*b*,*i*_, *E*_*b*, *i*_}; if process c itself were probed by the diathermal fiber, it would register another temperature not equivalent to that for processes a and b, given that processes a, b, and c are all in the steady state. How would one rationalize the above from a classical standpoint? We note that the apparatus for constructing the different ensembles pertain to equilibrium, possibly steady state NE situations (although there has been little work to establish the nature of these ensembles for even steady-state systems on a rigorous basis). The following assumes that the ensembles are valid for NE steady states. Define a phase space regional function *R*_*i*_, and a coordinate (*p*, *q*) set *Ω*_*j*_ where *R*_*i*_(*Ω*_*j*_) = *δ*_*ij*_, that is if the coordinates all belong to the region *R*_*i*_, then the function is unity, otherwise it is zero. Then, from the BQ construct, we can define a temperature T which applies to both thermostatic and steady state NE systems. Since steady state thermodynamical NE systems have extra variables to cater for gradients and fluxes of state variables, one cannot expect a direct correlation between thermostatic and the steady state NE system, as has been proved [22]. This is one reason why in the conventional description, distortions to the density-in-phase $\varrho \left(p,q\right)=Ce^{-\beta H}$ as in thermostatics with Θ = *kT*, *β* = 1/Θ for any one partitioned or composite process. For a composite system that is not uniform in temperature, but where the regions *i* have different temperatures, the density in phase implies for systems described well by the canonical distribution $\varrho_{i}\left(p\right)=C_{i}e^{\beta_{i}\sum_{j=1}^{m}\frac{p_{j}^{2}}{2m}}$ for a region $\left(p,q,t\right)\in \Omega_{i},t\in \Omega_{i}^{t_{i}}$, where $\int_{\Omega_{i}}\varrho_{i}\left(p\right)\text{dp}=1$, and $\int_{\Omega_{i}}\varrho_{i}\sum_{j=1}^{m}\frac{p_{j}^{2}}{2m}\text{dp}=\frac{m}{2}\Theta_{i}$. The total density with *n*_*p*_ temperature parameters would then have the distribution $\varrho \left(p,q\right)=\sum_{i=1}^{n_{p}}\varrho_{i}R_{i}\left(p,q\right)$ for a system with *n*_*p*_ temperature parameters. Regions *Ω*_*i*_ are all non-overlapping. The results presented in the concluding sections only assumes the validity of the Dhar-RLL condition Eq (30) for the definition of temperature in conjunction with the dithermal fiber.

Application of recoverable trajectories to heat conduction.

In developing the paradigm of heat conduction, only the k.e. of the localized particle along the lattice is relevant for the determination of temperature. Hence the classically exact Dhar-RLL equipartition result Eq (30) is of immediate relevance. We also write down the form that includes the harmonic potential to suggest possibilities for more complex dynamics than the model system presented here where Eqs (68–82) is not dependent on the potential form for the paradigm proposed, that of heat conduction being a recoverable trajectory and where the phenomena is subsumed by the Second law, which is a view not shared by BQ. The Dhar-RLL 1D result for *i* for a particle is

$$\begin{array}{c} \frac{1}{2}kT_{i}=<k.e.\left(i\right)>=<E_{i}-V\left(i\right)> \end{array}$$ (45)

where the *E*’s refer to energy; in statistical physics conventions, “heat” is taken to be the mean kinetic and potential energy of the system (p.212, Eq (6.4.3) [33]); only differentials of this quantity with a subtraction of the defined work differential terms yields the heat increment (p.214, Eq (6.5.9) [33]).

The temperature *T*_*i*_ of *i* from Dhar-RLL equipartition may be defined as an ensemble average, with *k* the Boltzmann factor as $T_{i}=\frac{2}{k}<k.e.\left(i\right)>$ where

$$\langle k.e.\left(i\right)+V_{i}\rangle \begin{array}{c} =\langle E_{i}\rangle . \end{array}$$ (46)

The above Eq (46) is computable for system in thermodynamical equilibrium. From the above, for any process *x* within a *x*-temperature characterization of a particle, with a quadratic or any arbitrary form of potential and where the kinetic energy also is present, we have as above $T_{i,x}=\frac{2}{k}<k.e.\left(i\right){>}_{x}$ and

$$\begin{array}{c} {\langle k.e.\left(i\right)+V_{i}\rangle }_{x}={\langle E_{i}\rangle }_{x}. \end{array}$$ (47)

The scattering processes must be defined. The particles would oscillate approximately about the mean position *q*_*i*_ = 0, and for any suitable choice of initial conditions, any process before a collision is termed process *b*, and *a* refers to a process commencing just after a collisional interaction of particle *i* and *i* + 1. The equilibrium distance between particles is set to 1 in reduced units. Let *pl*_*j*_ denote both the plane perpendicular to the vector *q*_*i*_ − *q*_*i*+1_ which contains the *q* coordinate point of contact between the particles during the *jth* collision of *i* and *i* + 1, and the *q* coordinate during this collision process. The collision would impart a change of velocity $\delta {\dot{q}}_{i}$ and kinetic energy *δk*.*e*.(*i*). By energy conservation, the average energy up to the time of collision for *i*, *Q*_*b*_ is (for any *i*)

$$\begin{array}{c} Q_{b}=\langle k.e.\left(i\right)+V_{i}{|}_{pl_{j}}\rangle \left(\text{averaged over all j collisions with i + 1}\right). \end{array}$$ (48)

We note that *Q*_*b*_ is also dependent on interaction with particle *i* − 1 which is not relevant here for the transfer energetics of *i* to *i* + 1. Primed variables refer to the quantity after a collisional interaction. For any *i*, *Q*_*b*_ is time independent when the system is in a steady state. Then the dynamical laws can be utilized to compute the change of k.e. for *i* (since the potential energy remains unchanged at *q*_*i*_ = *pl*_*j*_) and thus we can compute *Q*_*a*_ the energy just *after* the collision as

$$\begin{array}{c} Q_{a}=\langle k.e.^{\prime }\left(i\right)+V_{i}^{\prime }{|}_{pl_{j}}\rangle \left(\text{averaged over all j collisions}\right) \end{array}$$ (49)

where the time duration between the previous collision *j* − 1 is denoted Δt_*j*_; we define the mean time between *j* collisions as as *τ*_*mc*_. Averaging over the sampling time, we can compute average quantities such as the *Q*’s, denoted by brackets <> and so over time *τ*_*mc*_, the energy transfer *δw* is

$$\begin{array}{c} \delta w=<Q_{b}>-<Q_{a}> \end{array}$$ (50)

or *δw*/*τ*_*mc*_ per unit time over a continuous time period. Note that the normalized units used here (i.e. per unit time) are not the same as for the un-normalized work terms in Eqs (36 and 37). To be a comprehensive, we feature two forms of the 1-particle representation, one that assumes the equipartition result for harmonic potentials close to equilibrium assuming PLE and the other that relies on the Dhar-RLL definition of temperature that does not refer to the potentials at all.

For a harmonic potential, for a NE state close to equilibrium and which complies with the PLE, the following holds

$$\begin{array}{c} kT_{i,x}={\langle k.e.\left(i\right)+V\left(i\right)\rangle }_{x}={\langle E_{i}\rangle }_{x} \end{array}$$

where *x* ≡ *a* for processes just *after* a collision and *x* ≡ *b* for phases *before* a collision. The Dhar-RLL result allows us to write the following equations for any conservation potential $V_{c}$ which is not approximate but exact:

$$\begin{array}{ccc} k.e._{b,0} & = & k.e._{b}\left(l\right)+V_{c}\left(l\right)=E_{b}=Q_{b} \end{array}$$ (51)

$$\begin{array}{ccc} k.e._{a,0} & = & k.e._{a}\left(l\right)+V_{c}\left(l\right)=E_{a}=Q_{a} \end{array}$$ (52)

where *l* is the distance of the particle from the equilibrium plane (when *l* = 0, $V_{c}\left(0\right)=0$) during a collision and $V_{c}$ is the potential of the particle. After a collision, all the potential energy is converted to k.e. as the particle returns to *l* = 0 and the diathermal fiber is placed at the same location *l* = 0 to determine the temperature. Then taking averages (the bracketed quantities are averages) over all collisions, we can determine 〈*l*〉_*x*_ and also

$$\begin{array}{c} \frac{1}{2}kT_{b}=\langle E_{b}\rangle =\langle Q_{b}\rangle \end{array}$$ (53)

with a similar result for process *a*. Thus

$$\frac{\langle Q_{b}\rangle }{T_{b}}-\frac{\langle Q_{a}\rangle }{T_{a}}=0$$ (54)

solely as a result of the constancy of k for both the *a* and *b* processes. This result or any of the equivalents above is used as a template for creating a paradigm for continuous potentials. We note that this result is not at all dependent on any equipartition result that refers to the potential and the classically exact expression for temperature is used from the Dhar-RLL result. Furthermore, as long as a temperature is specified, there is no further reference to ergodicity as the zero entropy result is due to the subtraction of the same factors of *k* above, and in Eq (59) below, for instance. We note that the BQ diathermal fiber concept is used; if a steady temperature exists From Eq (46), for the simple case of harmonic potentials obeying equipartition, the following array of equations result:

$$\begin{array}{ccc} T_{b} & = & <E_{b}>/k=<Q_{b}>/k \end{array}$$ (55)

$$\begin{array}{ccc} T_{a} & = & <E_{a}>/k=<E_{b}-\delta w>/k \end{array}$$ (56)

$$\begin{array}{ccc} T_{a} & = & <E_{a}>/k=<E_{b}+\delta E>/k=<Q_{a}>/k \end{array}$$ (57)

$$\begin{array}{ccc} k & = & <\frac{Q_{a}}{T_{a}}>=<\frac{Q_{b}}{T_{b}}>. \end{array}$$ (58)

with *δE* = −*δw*. From one of the field properties of number theory (p.15, Field Axiom A4 [107]),

$$\begin{array}{ccc} k\;\;+\;\;(-k) & = & 0 \end{array}$$ (59)

we deduce

$$\begin{array}{ccc} \delta S=\frac{<Q_{b}>}{T_{b}}-\frac{<Q_{a}>}{T_{a}} & = & 0 \end{array}$$ (60)

which defines the recoverable trajectory. Some elementary remarks are in order concerning the averaging process. We note that for *N* (consecutive) samples

$$\begin{array}{c} <Q_{a}>=\left(\sum_{i=1}^{N}Q_{a,i}/N\right). \end{array}$$ (61)

Then from Eqs (59) or (60) and some *k*′ (here *k* = *k*′ for Newtonian mechanics involving masses),

$$\langle T_{a}\rangle =\frac{\sum^{\;}Q_{a,i}}{k^{\prime }N}=\frac{Q_{a}}{k^{\prime }}$$ (62)

$$\Rightarrow \;\frac{\langle Q_{b}\rangle }{\langle T_{b}\rangle }-\frac{\langle Q_{a}\rangle }{\langle T_{a}\rangle }=0$$ (63)

and the mean rate of energy transfer is *δw*/*τ*_*mc*_. This appearance of a rate coupled with the use of the BQ fiber for the determination of temperature provides the link between “thermostatics” and dynamics, although as argued here, the energy density function need not correspond to thermostatic densities. From the very basic equations above, a rate term can be deduced. The application of this recoverability transition in Eqs (68–82) requires a focus on the kinetic energy only, therefore all the potential terms may be are set to zero (or a constant):

$$V_{i}\;=\;V_{i}{|}_{pl_{j}}=V_{i}^{\prime }{|}_{pl_{j}}=0$$ (64)

and where

$$k\;=\;\frac{k_{B}}{2},\;=k^{\prime }=\frac{k_{B}}{2}.$$ (65)

### A generalization of the Zeroth law based on recoverable trajectory

The equilibrium understanding of the Zeroth law is that if two bodies are in diathermal contact, and there is no net exchange of energy over at least a characteristic time *τ*, then they are defined to be at the same temperature. The BQ definition of generalized temperature involving their diathermal fiber relies on the Zeroth law, using the energy approach. Another entropic approach is to examine the exchange of energy across the diathermal boundary between two systems 1 and 2 (these labels determine the heat and temperature of the systems) and if there is no net exchange of energy <*δQ*_1_ > = −<*δQ*_2_ > = 0, then if for any instant of time *δQ*_1_ = −*δQ*_2_ = 0 for non-work energy exchange only, and using the recoverable trajectory entropy change *δS* = 0, we have

$$\frac{\delta Q_{1}}{T_{1}}+\frac{\delta Q_{2}}{T_{2}}=\frac{\delta Q_{1}}{T_{1}}-\frac{\delta Q_{1}}{T_{2}},$$ (66)

$$\delta S=\frac{\delta Q_{1}}{T_{1}}-\frac{\delta Q_{1}}{T_{2}}=0\Rightarrow \delta Q_{1}\left(T_{2}-T_{1}\right)=0$$ (67)

or *T*_2_ = *T*_1_ if |*δQ*_1_| ≠ 0. Since in dynamic equilibrium |*δQ*_1_| ≠ 0 most of the time, we conclude *T*_2_ = *T*_1_, this time within the recoverable zero entropy formalism. Based on this same entropic formalism, one can define a system in transition to be in extended equilibrium if *δS* = *δ*(*Q*/*T*) = 0. Clearly a computable algorithm must be available for this extended concept. If we therefore define the Zeroth law in terms of Zero entropy transitions, then recoverable trajectories *δ*(*Q*/*T*) = 0 can conform to the Zeroth law even if their temperatures differ across a boundary if the criterion refers to zero entropy changes rather than energy (in at least closed systems). It will be shown that for such systems, these differing temperatures can be defined or at least calculated (as shown in the section below on applications of the theoretical reference model, especially Eqs (79–82)) For continuous systems in the 1-particle representation, such an association of two temperatures to a single particle (system) at different phases of its motion can be computed and Table 1 shows values of the second temperature *T*_*a*_; *T*_*b*_ in this case is <2*Q*_*b*_> with <*Q*_*b*_> given by Eq (73), where *T*_*b*_ is the standard particle kinetic temperature for the *b* process. In the current model of the continuous system consisting of single particle subsystems, the temperature is defined in terms of its kinetic energy where the particle *i* Hamiltonian coordinates are $\left({\dot{q}}_{i},q_{i}\right)$ with the potential energies partitioned away in our system definition Eqs (36 and 37). From the above discontinuous model, we note that the spatially stationary system—through stochastic averaging—consisting of the particle *i* is closed, since it always consists of one particle, but not isolated since it can exchange energy; in particular and it also conforms to a recoverable trajectory *δ*(*Q*/*T*) = 0 where the heat terms arises from the partitioned processes (*a* and *b*) where the diathermal fiber is used to determine temperature. If we therefore define the Zeroth law in terms of Zero entropy transitions, as above, then this closed system conforms to the Zeroth law, with regard to the energy exchange leading to a double temperature characterization for the particle for the partitioned states *a* and *b*; thus in this sense we can say that the two temperatures refer to the same particle in equilibrium with itself as defined from the extended Zeroth law.

We have theoretically demonstrated that subsystem (a) of Fig 1 must have the particle behaving as a recoverable trajectory based on the generalized Dhar-RLL equipartition theorem for 2 temperature, 1-particle subsystems. Hence if the above model is applied to a system that conforms to case (b) of the same Figure, and where there is numerical confirmation that the vector “‘work term’” $\delta \dot{w}\equiv \delta w/\tau_{mc}$ in unit time arising from recoverability theory is exactly equal to the energy transfer across the thermostated ends of the chain in unit time, then one can conclude that a verification of conductive heat as a recoverable process has been made. We demonstrate this to be the case for all the 9 atoms/particles situated evenly over the conductive lattice, where there is close numerical agreement between the independently determined heat transfer rate due to the thermostats and the numerical integration of various defined energy processes for all of the above labeled particles.

### Application of the Theoretical Reference Model described above to lattice particles interacting continuously via potentials

Here the results of the previous section for discontinuous energy interactions is applied to the problem of continuous steady state heat conduction (Fig 1(b)). This can be achieved by isolating the system to the “‘particle’” with coordinates $\left(q_{i},{\dot{q}}_{i}\right)$ and where with *k*/2 = *k*′, it follows that *k*′ < *T*_*i*_ > = < *k*.*e*.(*i*)_*max*_>. From Eq (53), we must have

$$\begin{array}{c} k^{\prime }\langle T_{i}\rangle =\langle k.e.\left(i\right)\rangle =\frac{1}{2}kT_{b}=\langle E_{b}\rangle =\langle Q_{b}\rangle \end{array}$$

This isolation is achieved by the conditions given in Eqs (64 and 65). For what follows the stochastic averages are determined, where the angle brackets <> is indicative of this process. The partial heat is defined as *Q* = *k*.*e*.(*i*) for *i*, where *k*′ *T*(*i*) = *Q* = *k*.*e*.(*i*). *T*(*i*) is then a type of fluctuating parameter associated with temperature. The reference system above is correlated to the current model of particles interacting with a continuous potential by realizing that each instant of time *t* involves an interaction via a potential, which takes the place of the transfer of energy due to elastic collisions and the conservation of momentum in the reference system. Thus for an interaction over time interval *dt*, and for the *j*th interval, the work performed on (*i* + 1) due to the force exerted by *i* is

$$\begin{array}{c} \delta w_{j}=F_{i+1,i}.\frac{dq_{i+1}}{dt}dt_{j}. \end{array}$$ (68)

Then $\frac{Q_{b}}{T_{b}}=k^{\prime }$ and after the interaction time *dt*_*j*_, the following results, where subscripts *a* and *b* refer to *after* and *before* the interaction over time interval *dt* at the *j*th interval respectively:

$$Q_{a}=\left(k.e.\left(i\right)-\delta w_{j}\right)$$ (69)

$$k^{\prime }T_{a}=\left(k.e.\left(i\right)-\delta w_{j}\right)=Q_{a}$$ (70)

and

$$k^{\prime }-k^{\prime }=0$$ (71)

⇒

$$\frac{Q_{b}}{T_{b}}-\frac{Q_{a}}{T_{a}}=\delta S=0$$ (72)

where Eq (72) defines a recoverable process. From the Theorem (p.177, “: If a generalized Carnot engine is disintegrating, then it is necessarily a frictionless device” [9]), we infer that for one-way scattering, such as for the thermal desorption problem, the Carnot optimum is always maintained because the stopping potential would convert the center of mass motion of the thermal packet into heat so that *δS* = 0. Eq (71) assures us that by virtue of that trivial equation, the thermal energy exchange for each consecutive collision *i* conserves the entropy term $S_{i}=Q_{i}/T$ so defined, and so the average would trivially also conserve the entropy no matter what the potential form. Thus energy transfer processes is controlled by the mediating potential which modifies the thermal energy content dynamically with time to maintain the mechanically irreversible Carnot optimized trajectory. It is important to bear in mind that our force fields have conservative potentials of the modified harmonic form Eq (35) where we have suitably partitioned the forces Eqs (36 and 37) acting on the particles to determine the work that will always ensure that Eq (72) obtains. Non-conservative potentials, that due for example to radiation of convection would obviously not allow for compliance. It is therefore important to model the equations to ensure that the fields are conservative, and therefore energy conserving, so that the First law may be applied directly to each consecutive step in the trajectory or else contrary results would ensue.

Then over *n* time intervals, where ΔT=ndt,

$$\begin{array}{c} <Q_{b}>=\int k.e.\left(i\right)dt/\Delta T. \end{array}$$ (73)

Define Δ**t** = *dt* and *F* = *F*_*i*+1,*i*_. For any one time interval Δ**t**, the loss of *k*.*e*.(*i*) which would give it some of the characteristics of *i* + 1, such as a lower temperature would also yield a heat content *Q*_*a*_ and work quantity *δw* given respectively by

$$\begin{array}{ccc} Q_{a} & = & k.e.\left(i\right)-F\frac{dq_{i+1}}{dt}dt_{j} \end{array}$$ (74)

$$\begin{array}{ccc} \delta w & = & \left(Q_{b}-Q_{a}\right)\;\;\;\;\text{spanning}\;\;\left[t_{1},t_{2}\right]. \end{array}$$ (75)

Over *n* time intervals, the averaged *nδw* has value

$$\begin{array}{ccc} n\delta w & = & \int_{t_{1}}^{t_{2}}k.e.\left(i\right)dt/\Delta t-\int_{t_{1}}^{t_{2}}\left(k.e.\left(i\right)-F\frac{dq_{i+1}}{dt}\Delta t\right)dt/\Delta t \end{array}$$ (76)

$$\begin{array}{ccc} & = & +\left(\int_{t_{1}}^{t_{2}}F\frac{dq_{i+1}}{dt}dt\right). \end{array}$$ (77)

Thus *δw* *per unit time* is given by

$$\begin{array}{c} \left(\int_{t_{1}}^{t_{2}}F\frac{dq_{i+1}}{dt}dt\right)/n\Delta t \end{array}$$ (78)

which is defined as being identical to *δw*_1→2_ in Table 1 and Eq (39). There is therefore complete concordance between the two models. Here energy transitions are modeled in terms of one particle that conforms to a recoverable process. Relative to the work transitions, one might wish to also characterize the lower exchange temperature and heat energy *Q*_*a*_ that allows for work to be performed on the adjacent elements. Over *n* time intervals *dt*, one has

$$\begin{array}{c} <Q_{a}\left|{}_{n}\right.>=\frac{\int_{t_{1}}^{t_{2}}\left(k.e.\left(i\right)-F\frac{dq_{i+1}}{dt}\Delta t\right)dt}{\Delta t}. \end{array}$$ (79)

Then <*Q*_*a*_> for one time interval is

$$\begin{array}{c} <Q_{a}>=\frac{\int k.e.(i)}{n\Delta t}-\left(\frac{\int F\frac{dq_{i+1}}{dt}dt}{n}\right). \end{array}$$ (80)

Hence,

$$\begin{array}{ccc} <Q_{a}> & = & \left\{<Q_{b}>-\delta w_{1\to 2}.dt\right\}=\alpha \end{array}$$ (81)

$$\begin{array}{ccc} & \text{and} & <T_{a}>=2\alpha . \end{array}$$ (82)

The results for <*T*_*a*_> are provided in Table 1. We observe that it is lower, as it should be, and this temperature is associated with the particle *i*. Thus in any one location, we observe that we can evoke 2 temperatures that are consistent with the energy transfer across the crystal and the extended Zeroth law in this modeling scheme.

## Results

The use of an anharmonic potential allows for the system to exhibit a near linear temperature gradient as shown in the snapshot in Fig 2 of a typical run [54–58]. The details of the simulations follow below.

All fluctuations in quantities are expressed as the uncorrected standard deviation *u.s.d*. and the error ± are expressed in terms of this *u.s.d*. The *u.s.d*. is the ordinary standard deviation *σ* where $\sigma =\sqrt{\frac{1}{N}\sum_{i=1}^{N}{\left(x_{i}-\mu \right)}^{2}}$ where the mean *μ* is $\mu =\frac{1}{N}\sum_{i=1}^{N}x_{i}$; *x*_*i*_ are the experimental values with *N* sample points. The E notation represents exponents to base 10. The results are for the following parameters:

$$\begin{array}{c} k_{h}=1.0,b_{h}=0.5\left(\text{eq}.35\right),d_{eq}=1.0 \end{array}$$

for the equilibrium interparticle distance *d*_*eq*_ set to 1 reduced units, where the potential is zero. After many successive equilibration runs amounting to about 500M timesteps (where *dt* = 0.001 for the timestep in reduced units), the production runs were initiated. The numerical integrations utilized the well-established symplectic Velocity Verlet algorithm of Swope, Anderson, Berens and Wilson (p.81, Eqs 3.17–3.21 for the Allen et al. reference [108, 109]), which is essentially second order. The production runs are for 100M timesteps, where a continuous sampling of 3M time-steps (constituting a dump) are made over 20 dumps, where for each dump, averages are made over the 3M time-steps. The various statistics are obtained over another average over these 20 dump values and the fluctuations expressed as the uncorrected standard deviation (*u.s.d*.). A current vogue in these studies is the fifth-order Runge-Kutta integrator algorithm [55], where it is arguable whether more accurate results necessarily obtain due to machine error accumulation; the objective here is to establish some principles that require sampling a relatively larger portion of phase space that would be precluded by computational costs of more intensive computational algorithms that are only relevant for accurate determination of specific properties over a smaller region of the phase space volume.

The rate of energy transfer into the 200 hot thermostated atoms at the left hand side of the system is 0.21747E+00 ± 0.34577E-01 and for the 200 colder atoms is -0.21229E+00 ± 0.12843E-01. We find that the energy transferred to the adjacent atoms in Table 1, *δw*_1→2_ based on recoverability theory is in excellent quantitative agreement to the independently determined energy flow into the thermostats in every instance. We note that the reverse work of particle *i* + 1 → *i* is negative meaning that there is a gain of energy due to its own force field. The important point therefore is that one is observing a type of *net* one way scattering of energy, from *i* to *i* + 1 due to the conservation of energy because atom *i* − 1 would have to scatter energy into *i* to compensate for the loss of energy through the force field on the work done to *i* + 1.

## Discussion and Conclusion

Over especially the last two centuries, the main developments in thermodynamics include (a) the partitioning of the energy into two distinct forms, work *W* and heat *Q*, from which the entropy is derived from the latter where the entropy differential *dS* for a closed system is *dS* = *dQ*/*T*, and (b) where the field of statistical theory in concerned, these concepts were cast in terms of the Liouville equation and the associated Hamiltonian for both classical and quantum systems. The mathematical contradictions of both the Liouville equation and the time reversibility assumptions, both of which were incorporated into the more modern versions of thermodynamical theories [97, 110] have been demonstrated before [7, 17, 66, 67]. Here we have outlined a method of conflating both these quantities via the concept of recoverable transitions by creating a single particle model that incorporates all primitive interactions associated with recoverability. Thus within this paradigm, the classical understanding of “heat” as that form of energy that is transferred as a result of a temperature difference (due to the different source temperatures) may be viewed in a recoverable trajectory paradigm as a work term, where such an association is strictly forbidden within the conceptual structure of standard thermodynamics. By extending the reference model to a continuous system, it was shown from the simulation results that a paradigm exists which points to the possibility of framing mathematical models and equations that conforms to a recoverable trajectory. The paradigm developed above provides the boundary conditions and overall structure for the solution of differential and integral equations to arbitrary accuracy by distinguishing interactions by partition schemes, leading in the examples above of different temperatures associated with the same region or particle body, and where the probability distribution profiles for processes a and b differ due to temperature differences and energy densities. The paradigm also yields a ‘net effect’ of NE distribution profiles which is the standard model in the conventional descriptions in NE theory. Here the feasibility of the paradigm is illustrated by the partitioning schemes due to a method of distinguishing different types of processes in this process of deconvolution.

Of great interest, and of great challenge, is to extend these models to more than one particle, so that the kinetics and thermodynamical profile of the system might be computed from these elementary considerations, for instance to fluid systems. We also contradicted the view that the Fourier law is strictly a locally defined process, unrelated to the Second law or which cannot be deduced from the Second law, a claim central to the work of BQ in their theory of thermoelectric and thermomagnetic effects [5], by framing elementary theoretical propositions that are then tested out numerically in simulations for their feasibility, which incorporates the Second law locally. BQ interpreted the Second law as pertaining to globally coupled heat-work transitions, whereas (Fourier) heat conduction was viewed as local and also as only one component of the energy definition (that of heat), and therefore had only local significance unrelated to the Second law.

The Clausius, Kelvin, and more recently Planck and Carathéodory statements of the Second law all necessitated the construction of pathways that were adiabatic or isothermal in nature in order to deduce a global optimized value of the work that can be performed in a cycle. The conflating of the Carnot cycle into a single particle interaction attempted here may imply the possibility of a corresponding extension of the axiomatic basis of thermodynamics to encompass some of the transformations described here, e.g. the transfer of energy as “heat” energy across the lattice chain to the thermal reservoirs which is locally a “work” term in the 1-dimensional representation, and more importantly, for processes that are non-cyclical; the traditional renderings of the Second law required stationary cycles, and the BQ view of **F** lent some credence to this development.

One extension attempted here is the Zeroth law, where two temperatures may be associated with a single particle in a classically considered irreversible system. In the 1-particle scheme outlined here, it is possible to structure equations that represents the key quantities {*Q*_*a*_, *Q*_*b*_, *δw*, *T*_*a*_, *T*_*b*_} associated with the conflated Carnot engine, which represents the most basic reduction unit. The next stage would be to consider what are the time dependent processes expressed in mathematical terms that can contribute to the above 5 primitive variables, in terms of local fluxes, forces and their various conjugate variables, especially the gradients. Such a characterization would solve or simplify the local problem of description. By analogy, extending these ideas over more than one particle could conceivably lead to non-local descriptions of the various kinetic phenomena.

It seems feasible from the preliminary results obtained here that thermodynamical processes, both equilibrium and otherwise may be expressed in terms of a single concept where equilibrium and NE processes might appear as limiting cases of this singular interpretation. What is of interest in these initial forays is the nature of the representation; here we are able to model systems that seem to conform to recoverable trajectories for single particle interactions. Questions that immediately suggest themselves are whether these models are unique, and if not, do they imply a multiplicity of modes that recoverability theory can accommodate to interpret thermodynamical phenomena relative to the unified concept being proposed here. Other interesting questions would be to elucidate the relationship between the Carathéodory development of the Second law concerning the non-accessibility of certain adiabatic states by isothermal transformations in the equilibrium state and how this situation arises from the above NE development.
